# Single Particle Nanothermometry Quantifies Heating in a Plasmonic Trap

**DOI:** 10.1021/acsnano.6c03979

**Published:** 2026-08-13

**Authors:** Fengchan Zhang, Manuel Romero, Khouloud Hamraoui, Jiachen Zheng, Thi Tuyen Ngo, Ginés Lifante-Pedrola, Gabriel Lozano, Hernán Míguez, Fan Wang, Jorge Rubio-Retama, Daniel Jaque, Patricia Haro González

**Affiliations:** † Nanomaterials for Bioimaging Group (nanoBIG), Departamento de Física de Materiales, Facultad de Ciencias, 16722 Universidad Autónoma de Madrid, Madrid 28049, Spain; ‡ Instituto Nicolás Cabrera (INC), Facultad de Ciencias, Universidad Autónoma de Madrid, Madrid 28049, Spain; § Spanish National Research Council, 201472 Institute of Materials Science of Seville, University of Seville, Seville 41092, Spain; ∥ MatNaBIO Research Group, Department of Chemistry in Pharmaceutical Sciences, Faculty of Pharmacy, Complutense University of Madrid, Madrid 28040, Spain; ⊥ School of Physics, 12633 Beihang University, Beijing 100191, People’s Republic of China

**Keywords:** plasmonic enhancement, thermoplasmonics, optical tweezers, upconverting nanoparticle, nanothermometer

## Abstract

Plasmonic trapping enables nanoscale confinement of individual nanoparticles by exploiting localized electromagnetic field enhancement, an effect inherently accompanied by photothermal heating. Quantifying this local temperature increase is essential, as heating can affect trap stability through enhanced Brownian motion and the emergence of thermally induced forces such as thermophoresis. Here, we experimentally determine the local temperature experienced by a single upconverting nanoparticle optically trapped in the vicinity of a pyramid-like gold nanoantenna. By combining this measurement with numerical simulations of the heat distribution, we infer the temperature at the gold–water interface. While the trapped UCNP experiences a temperature increase of ∼40 °C at an average distance of ∼50 nm from the nanoantenna surface, thermal simulations predict that the nanoantenna surface may reach temperatures of up to ∼100 °C under the same excitation condition. Analysis of the nanoparticle’s luminescence provides simultaneous determination of its local temperature and three-dimensional motion in the vicinity of the nanoantenna. The results reveal a linear increase in temperature with trapping power and demonstrate that the particle explores regions characterized by strong thermal gradients generated by the plasmonic structure. By correlating temperature and position, we estimate the relative magnitudes of optical and thermophoretic forces, providing a model-based explanation for the observed steady-state trapping position. These findings establish individual upconverting nanoparticles as probes for investigating thermoplasmonic effects at the single-particle level and provide insight into the interplay between optical confinement, thermal gradients, and Brownian motion in plasmonic trapping systems.

## Graphical abstract

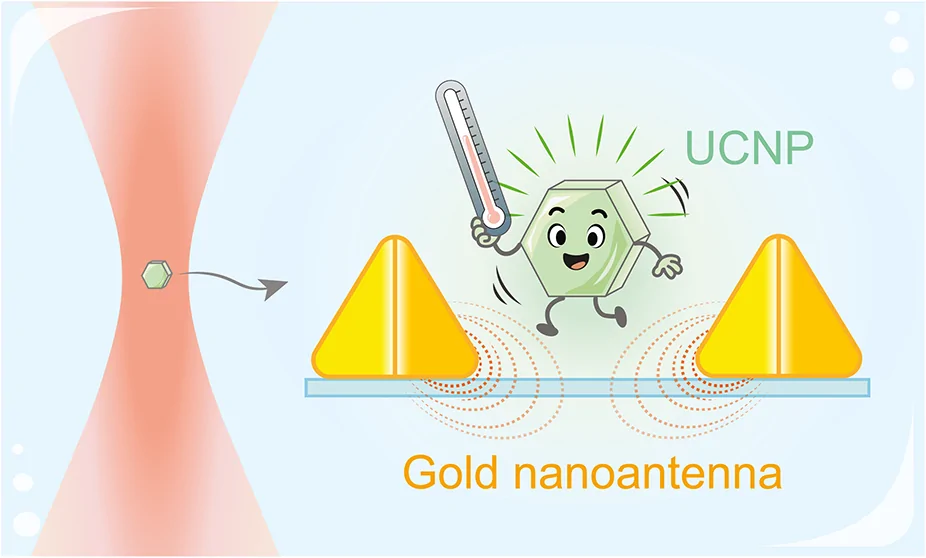

## Introduction

Plasmonic nanoantennas are metallic nanostructures designed to support localized surface plasmon resonance (LSPR), which confine electromagnetic fields into subwavelength volumes.^1^ When illuminated at or near their resonant wavelength, these structures generate intense near-field enhancements that can be exploited for the optical trapping and manipulation of colloidal nanoparticles.^2–5^ In particular, plasmonic nanoantennas have been used to confine luminescent nanoparticles or molecules that are only weakly trapped in conventional optical tweezers.^6–8^ However, the same mechanisms that enhance the local field also lead to significant photothermal heating.^9^ Local temperature increases around plasmonic nanostructures have been found to be beneficial for several applications, including thermal therapy,^10–12^ drug delivery,^13,14^ and the control of fluid flows in microfluidic devices.^15,16^

For plasmon-assisted trapping at the single-particle level, however, local heating emerges as a critical parameter influencing the trapping performance. Temperature rises of several tens of degrees can induce convection currents, alter the local density of the medium, enhance Brownian motion, and trigger thermally driven fluid instabilities, all of which compromise the stability of the optical trap. Moreover, the temperature field around plasmonic antennas exhibits a rapid spatial decay that gives rise to steep thermal gradients in the vicinity of the antenna. These gradients can generate additional forces such as the effective thermophoretic force, which may be comparable to the optical force and thus shift or destabilize the trapping position.^17–19^ For these reasons, it is essential to quantify the temperature reached by a plasmonic nanoantenna within an aqueous trapping experiment and to define precisely where this temperature is evaluated with respect to the antenna. This is particularly crucial when aiming to trap luminescent nanoparticles in the vicinity of antennas to enhance their emission down to the single-particle level, enabling sensing applications and the study of nanoscale dynamics.

Measuring temperature at the nanoscale in aqueous environments presents considerable challenges. Among the different experimental approaches under development, luminescence nanothermometry has emerged as a promising route. This methodology relies on nanoparticles whose emission properties show a well-defined dependence on temperature. A widely used class of luminescent nanothermometers is upconversion nanoparticles (UCNPs), typically NaYF_4_ doped with Yb^3+^ and Er^3+^ ions.^20,21^ Their thermally coupled electronic levels enable temperature determination through ratiometric analysis of visible emission bands under near-infrared excitation. Beyond their use as nanothermometers, UCNPs have been extensively explored in biosensing,^22,23^ bioimaging,^24,25^ and photodynamic therapy.^26^ In combination with optical tweezers and single particle spectroscopy, they have enabled physiological sensing at the cellular level,^27–30^ force sensing,^31^ chemical sensing,^32^ and investigation of plasmonic enhancement at the single-particle level.^33,34^

Previous approaches to temperature measurements in plasmonic trapping systems have provided important information, but they usually probe either the temperature of the plasmonic structure itself or an averaged temperature in its surrounding environment. Anti-Stokes emission, Raman-based methods, nanopore ionic currents, fluorescence intensity, fluorescence lifetime, and diffusion-based approaches have been used to quantify plasmonic heating. UCNPs have also been used as local thermometric probes in plasmonic trapping systems.^48^ However, in this previous experiment, the thermometric readout was not combined with an experimental determination of the precise position of the UCNP with respect to that of the plasmonic structure. This limitation is particularly relevant because both the optical near-field and the temperature field decay over nanometric distances from the metallic nanostructure. Therefore, a local temperature cannot be fully interpreted without knowing where the probe is located within the nanotrap.

In this work, we go beyond temperature measurement alone by combining single-particle upconversion thermometry with fluorescence-assisted three-dimensional tracking of an individual optically trapped UCNP. This enables the simultaneous determination of the local temperature experienced by the nanoparticle and its position with respect to the plasmonic nanoantenna. Our approach, therefore, extends previous studies by providing, for the same individual nanoparticle, simultaneous access to three-dimensional motion and thermal sensing, allowing us to explore optical confinement, luminescence enhancement, local heating, thermal gradients, and thermophoretic effects at the single-particle level under aqueous trapping conditions.

## Results and Discussion

The gold nanoantenna array (GNA) investigated in this work consists of gold nanopyramids fabricated on a glass substrate. The structures were produced by a microbead-assisted method detailed in Section S1 in the Supporting Information.^35,36^ In brief, an ordered monolayer of nanospheres was deposited on glass, followed by gold evaporation. Gold accessed the substrate through the intersphere gaps, resulting in a large-area hexagonal array of nanopyramids (Figure 1a). The statistical analysis of the scanning electron microscopy (SEM) images reveals an interpyramid distance of 190 nm and an effective side length of 140 nm (Figure 1b). The corresponding extinction spectrum obtained from electromagnetic simulations indicates that the LSPR is far from the 980 nm excitation wavelength (Figure 1c). This prevents the formation of bubbles under tightly focused 980 nm laser irradiation, which would otherwise compromise the optical-trapping experiment.^33^ Despite this spectral mismatch, electromagnetic simulations reveal that focusing a 980 nm beam onto the GNA generates hotspots at the pyramid vertices (Figure 1d and Video S1), which correspond to the expected trapping location of a colloidal UCNP near the GNA. Numerical simulation at 550 nm (the emission wavelength of our UCNPs) reveals that field enhancement is localized on the edge of the gold pyramids rather than at the vertices, with an overall lower enhancement magnitude (Figure 1e). Therefore, the emission enhancement caused by the Purcell effect is not expected, since the particle is trapped at a spatial location (vertex) where field enhancement at the emission wavelength is not produced.

**Figure 1:**
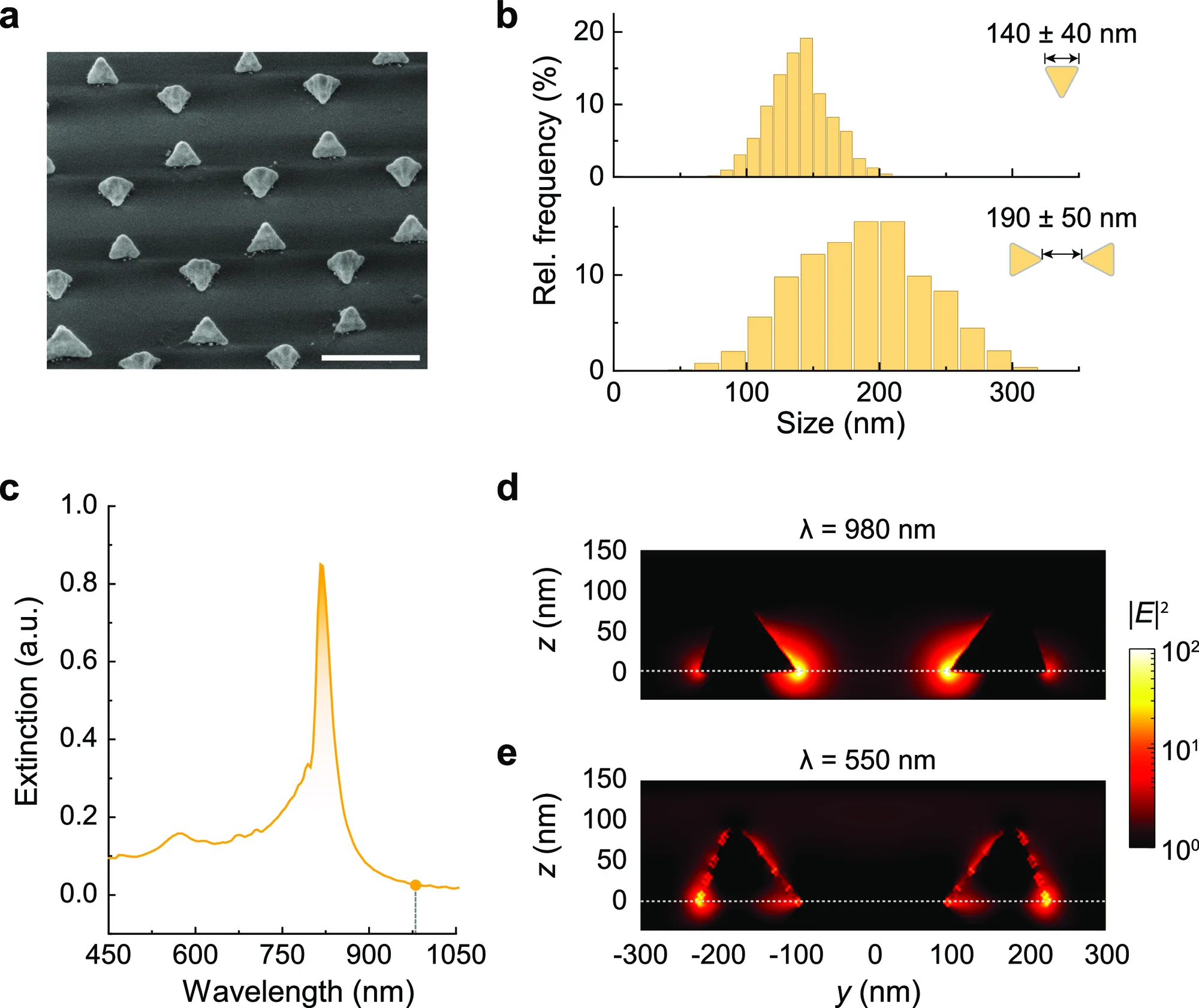
Characterization of the gold nanoantenna array. (a) Representative SEM image of the array of pyramid-like gold nanoantennas. Scale bar: 500 nm. (b) Size distribution of more than 500 pyramid-like nanoantennas extracted from the analysis of the SEM images. (c) Extinction spectrum obtained from electromagnetic simulations. (d) Simulated near-field intensity distribution (cross-sectional view) of the 980 nm excitation for a pyramid-like nanoantenna array. (e) Simulated near-field intensity distribution (cross-sectional view) at 550 nm (overlaps with the emission of the UCNP) for a pyramid-like nanoantenna array.

For plasmonic thermometry, we employed hexagonal NaYF_4_ nanoparticles codoped with Er^3+^/Yb^3+^ ions with an average size of 30 nm (Figure 2a,b). Their synthesis procedure is described in Section S2 in the Supporting Information. Under 980 nm excitation, the UCNPs exhibit the characteristic green upconversion bands arising from the ^2^H_11/2_ → ^4^I_15/2_ and ^4^S_3/2_ → ^4^I_15/2_ transitions (Figure 2c). While these emissions are nominally two-photon processes and thus expected to scale quadratically with excitation power, under the high intensities required for optical trapping (on the order of MW·cm^–2^), we observe a sublinear dependence, with the degree of saturation becoming more pronounced as the excitation power density increased (Figure 2d), consistent with saturation of intermediate and emitting states at large excitation rates.

**Figure 2:**
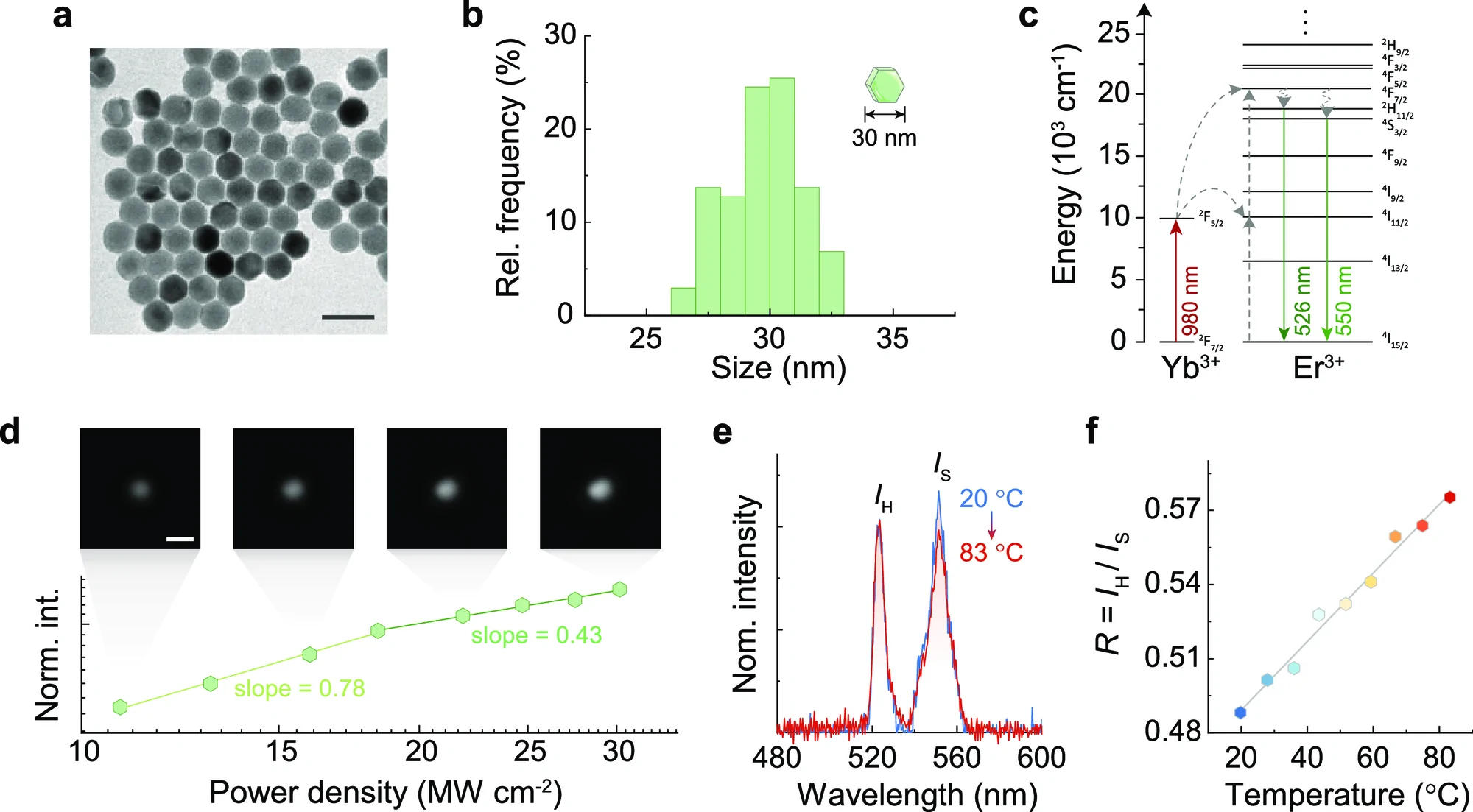
(a) Representative transmission electron microscopy (TEM) image of UCNPs used in this work. Scale bar: 50 nm. (b) Size histograms obtained from more than 200 UCNPs in TEM images. (c) Energy-level diagram corresponding to Yb^3+^ and Er^3+^ ions. The excitation (980 nm) and transitions associated with two green emission bands are indicated. (d) Top panel: Luminescence images generated by UCNPs under 980 nm excitation with different power densities (10, 16, 22, and 30 MW cm^–2^) obtained at 20 °C. Bottom panel: Variation of the luminescence intensity as a function of the excitation power density at 980 nm. Scale bar: 3 μm. (e) Emission spectra generated by UCNPs under 980 nm excitation as obtained at two different temperatures, 20 and 83 °C. (f) Temperature dependence of the intensity ratio *R* as obtained from the emission spectra recorded from 20 to 83 °C.

Because the ^2^H_11/2_ and ^4^S_3/2_ levels are thermally coupled, the emitted intensities corresponding to the ^2^H_11/2_ → ^4^I_15/2_ (*I*_H_, 520–535 nm) and ^4^S_3/2_ → ^4^I_15/2_ (*I*_S_, 535–560 nm) transitions are correlated, making the intensity ratio *R* = *I*_H_/*I*_S_ a reliable thermometric parameter.^20^ Emission spectra recorded at two different temperatures under an excitation of 20 MW·cm^–2^ (Figure 2e) reveal a decrease in the relative contribution of the ^4^S_3/2_ → ^4^I_15/2_ emission band. This trend leads to a monotonic increase in the intensity ratio *R* with rising temperatures (Figure 2f). From *R* vs *T* experimental data, it is possible to determine the relative thermal sensitivity (*S*_r_) of our UCNP, defined as *S*_*r*_ = *R* ^–1^·d*R*/d*T,* yielding a value of *S*_r_ ≈ 0.3%·°C ^–1^ at 20 °C. This relative thermal sensitivity is lower than typical values reported for Yb^3+^/Er^3+^ UCNPs under low-power excitation (typically around 1%·°C^–1^).^37^ This reduction in relative thermal sensitivity is consistent with previous studies, which attribute sensitivity loss to the growing influence of multiphonon relaxation in the high-power regime, as it has been experimentally determined in Section S3 of the Supporting Information.^38^ The *R* vs *T* calibration curve (Figure 2f) allows for the determination of increment in the temperature of the UCNP (Δ*T*) from the experimentally determined increment in the intensity ratio (Δ*R*) as $\Delta T\left(\mathrm{{}^{\circ}C}\right)=\frac{\Delta R}{R}\frac{1}{0.003}$ .

To perform thermal measurements at the nanoantenna, a microfluidic chamber was assembled on the glass substrate containing the array of nanoantennas. The dimensions and position of the microchamber were optimized to be partially covered by the GNA. This configuration enables a single optically trapped UCNP to be scanned between the bare glass substrate and the GNA-covered region. The chamber was filled with aqueous UCNP dispersion. The concentration of the aqueous UCNP dispersion is reduced to 10^–7^ mg/mL to ensure single particle trapping (Section S4 in the Supporting Information). Although several nanoantennas are illuminated within the approximately 1 μm diameter excitation spot, the large mean interparticle distance (>100 μm) in the highly diluted dispersion makes simultaneous trapping of more than one UCNP highly unlikely. In addition, each experiment was initiated by trapping a single UCNP on the bare glass region and subsequently translating the same particle toward the GNA-covered region. For single UCNP trapping and manipulation, a single 980 nm laser beam was tightly focused into the chamber using an oil-immersion microscope objective (100×/NA 1.4). The optical setup enables simultaneous optical trapping, single-particle luminescence imaging, and the acquisition of the emission spectrum generated by the optically trapped UCNP (Figure S3). The analysis of the acquired luminescence images allows for three-dimensional particle tracking (see more details in the Methods section). The trapping dynamics differ markedly between glass and GNA-covered substrates (Video S2 in the Supporting Information). On bare glass, the UCNP luminescence spot fluctuates in size and position (Figure 3a), reflecting substantial particle delocalization. In contrast, in static conditions (substrate and 980 nm beam are fixed), trapping on the GNA yields a stable luminescence signal (Figure 3b), indicative of enhanced field confinement. When the 980 nm laser beam is scanned over the patterned region, the UCNP can undergo discrete transitions between neighboring antenna hotspots (Section S6 and Video S3). Single-particle 3D trajectories in static conditions were reconstructed from luminescence videos using spot size variation for axial (*z*) (Figure S6) localization and centroid position for *x*–*y* tracking. The three-dimensional single-particle trajectories (Figure 3c,d for a single UCNP trapped on bare glass and on GNA, respectively) demonstrate a pronounced improvement in confinement near the nanoantenna. The projected *x*–*y* trajectory (Figure 3e,f) shows a reduction in positional fluctuation (*x*–*x*_eq_) = (*y*–*y*_eq_) from 90 nm down to 70 nm (*x*_eq_ and *y*_eq_ are the equilibrium position along *x* and *y* axes, respectively) when moving from the glass to the GNA, corresponding to an increase in the effective trap stiffness from 0.3 to 0.5 pN·μm^–1^ (see calculations in Section S7). Note that the projected trajectory on the substrate with GNAs is displaced from the beam center. This positional shift further indicates an antenna-assisted trapping effect, as the hotspot is located near the vertex of the GNAs, and it may not coincide with the center of the beam (Figure S5). Axial position histograms corresponding to a single UCNP optically trapped on the bare glass substrate and on the GNA (Figure 3g,h) also reveal a nanoantenna-induced reduction in the axial positional fluctuation (from (*z*–*z*_eq_) = 45 nm on the glass substrate down to (*z*–*z*_eq_) = 22 nm on the GNA). Details on how axial position histograms were obtained from the luminescence videos are given in Section S8 of the Supporting Information. The uncertainty in the axial position was evaluated by propagating the fitting uncertainty of full width at half-maximum (FWHM) of the luminescence-spot through the simulated FWHM–*z* calibration curve. This analysis yields an axial uncertainty of approximately ±10 nm at *z* ≈50 nm. The complete localization procedure and uncertainty analysis are provided in Section S8 of the Supporting Information. Furthermore, the axial position histograms also reveal a clear displacement of the mean particle position from ∼120 nm above bare glass substrate down to ∼50 nm above the GNA. This axial shift, together with the stronger confinement, is consistent with the strong enhancement of the 980 nm field at the substrate interface (Figure 1d), which pulls the UCNP closer to the nanoantenna surface. A possible source of uncertainty in the localization of UCNPs near the plasmonic nanoantenna is the antenna-induced modification of the emission pattern, which could introduce a small bias in centroid-based position determination. Although the UCNP emission overlaps spectrally with the nanoantenna optical response, simulations show that the emission-wavelength field has limited overlap with the UCNP trapping position (Section S9 in the Supporting Information). In addition, the calculated Purcell factor remains close to unity in the region where the UCNP is located. Therefore, the stiffness values obtained from positional fluctuations near the nanoantenna (provided later in the paper) should be regarded as effective values, potentially including a minor contribution from localization bias.

**Figure 3:**
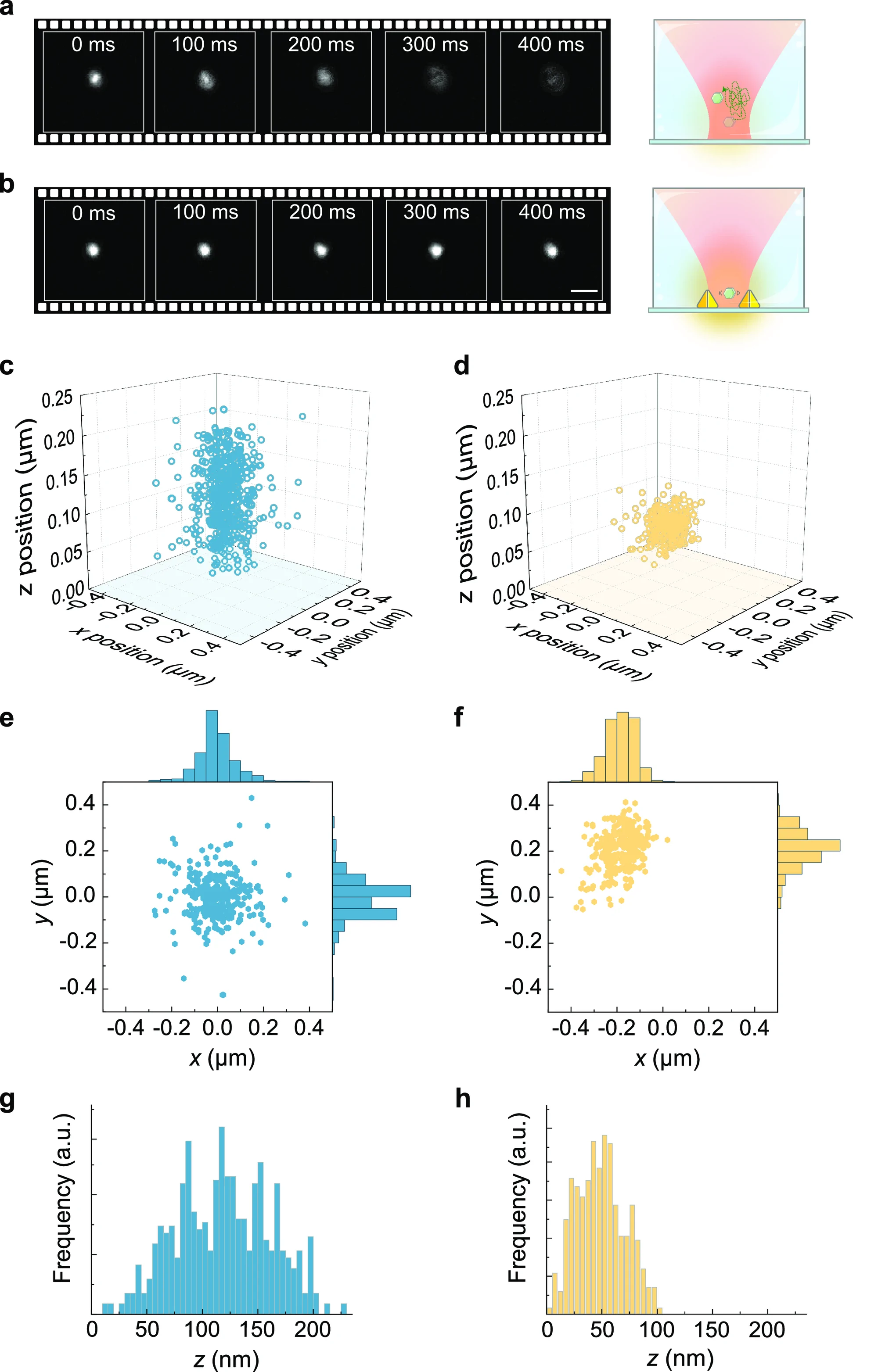
Comparison of the particle trajectories on bare glass and GNA-covered substrate. (a) Left: Sequence of luminescence images corresponding to a single UCNP optically trapped on bare glass. Note that the sequence shown here does not represent a complete cycle of size variation. The actual changes occur on a much shorter time scale, and multiple cycles may have occurred within the long interval between frames. Right: schematic diagram of the Brownian motion of a single UCNP trapped on the glass. (b) Left: Sequence of luminescence images corresponding to a single UCNP optically trapped on a GNA-covered substrate. Right: schematic diagram of the positional fluctuations of a UCNP trapped in the vicinity of the gold nanoantennas, where the fluctuations are significantly reduced relative to those observed on the glass. Scale bar: 2 μm. Three-dimensional trajectory of a single UCNP optically trapped on (c) bare glass and (d) a GNA-covered substrate. *x*–*y* trajectory of an optically trapped UCNP on (e) bare glass and (f) a GNA-covered substrate. Axial position histograms corresponding to an optically trapped UCNP on (g) bare glass and (h) a GNA-covered substrate.

The luminescence image sequences (Figure 3a,b) demonstrate not only a more stable confinement of the particle on the GNA-covered substrate but also a marked enhancement in upconversion emission intensity. Analysis of luminescence images from different UCNPs reveals an increase in the luminescence intensity when the particle is trapped on the nanoantenna, compared with trapping on bare glass. This enhancement is first confirmed by measuring the emitted intensity generated by an optically trapped UCNP when driven from the bare glass to the GNA (Figure 4a). The intensity–time profile reveals that the emission intensity of the particle exhibits a 2-fold enhancement upon being trapped within the nanoantenna array (Figure 4b). The emission enhancement factor, β = *I*_GNA_/*I*_G_ (where *I*_GNA_ and *I*_G_ are the emitted intensities of the UCNP measured on the GNA and on the glass, respectively), was found to be particle-dependent. By repeating the experiments shown in Figure 4b on different individual UCNPs, we obtained an average enhancement factor of β = 2.3 ± 0.6. The nanoantenna-induced emission enhancement is also confirmed by spectrally resolved measurements, which show a pronounced increase in the green emission band (Figure 4c). A similar analysis performed on the spectra of individual UCNPs reveals an average luminescence enhancement factor of β = 2.3 ± 0.8 (Figure 4d), in reasonable agreement with the value obtained from the luminescence images. The moderate enhancement factors observed here should be interpreted in the context of a dynamically trapped colloidal nanoparticle. Larger enhancement factors can be achieved in optimized bowtie, nanohole geometries, or nanocavity, particularly in immobilized or dry configurations where the emitter–antenna distance and orientation are fixed.^39–41^ For example, UCNP thin films coupled to colloidal-lithography nanoantenna arrays have shown enhancement factors up to 20–35× under static conditions.^42^ In contrast, in our experiments, the UCNP remains a freely fluctuating nanoparticle in an aqueous environment, so the coupling to the nanoantenna is transient and affected by Brownian motion, near-wall dynamics, thermal gradients, and thermophoretic effects. The observed 2–4× enhancement is, therefore, sufficient to reveal plasmon-assisted confinement and excitation-field amplification under realistic liquid-phase trapping conditions. As discussed previously, the nanoantenna-induced luminescence enhancement is attributed to the local enhancement of the 980 nm electric field. We recall here that the numerical simulations indicate that the UCNP particle is trapped at the vertex of the gold nanopyramid where the 980 nm local intensity is maximized. The emission enhancement due to the Purcell effect is disregarded because the amplification of 550 nm radiation is produced at the edge of the gold nanopyramid, without overlapping with the UCNP location (Figure 1e). This interpretation is further supported by the calculated Purcell factor, which remains close to unity at emitter–antenna separations larger than approximately 20 nm, whereas the excitation-field intensity enhancement at 980 nm remains significant, reaching approximately 5.6 at a distance of 20 nm from the antenna tip. Therefore, within the spatial region sampled by the trapped UCNP, excitation-field amplification dominates radiative-rate modification. Further details are provided in Section S9 of the Supporting Information. Figure 1d reveals that the 980 nm field intensity at the vertex of the nanoantenna can be enhanced by nearly 2 orders of magnitude. This value is significantly larger than the average experimentally observed luminescence enhancement. Two effects help explain this discrepancy. First, rather than being trapped exactly at the antenna vertex, the UCNPs are situated at an average vertical offset of ∼50 nm (Figure 3h). Second, the UCNP emission scales sublinearly with excitation power (Figure 2d). From the simulated 980 nm intensity map, we extract the amplification profile along the axial direction (Figure 4e). When we overlap this profile with the experimentally obtained axial-position histogram, it becomes evident that the UCNP experiences excitation intensities substantially lower than the maximum localized at the vertex (Figure 4e). By combining the simulated field profile with the measured axial distribution, we obtain a histogram of the expected excitation-intensity amplification (Figure S10), from which we estimate an average amplification factor of ∼4. The 980 nm power density at the focus of the Gaussian beam when not interacting with GNA is 4 MW cm^–2^. Thus, considering an average amplification of 4 leads to, in a first-ordered approximation, a local 980 nm power intensity of 16 MW cm^–2^ when performing optical trapping on the GNA array. For a 980 nm power intensity of 16 MW cm^–2^, the UCNP emission intensity scales approximately with the excitation intensity as *I* ^0.8^ (Figure 2d); this average field amplification yields a predicted luminescence enhancement of 4^0.8^ ≈3. This value is in good agreement with the experimental results. Thus, we conclude that the enhancement in UCNP emission intensity is primarily caused by the local amplification of the 980 nm excitation field at the vertex of the gold nanopyramid. The magnitude of this enhancement is constrained by the sublinear power dependence of the upconversion emission and the spatial delocalization of the UCNP relative to the hotspot.

**Figure 4:**
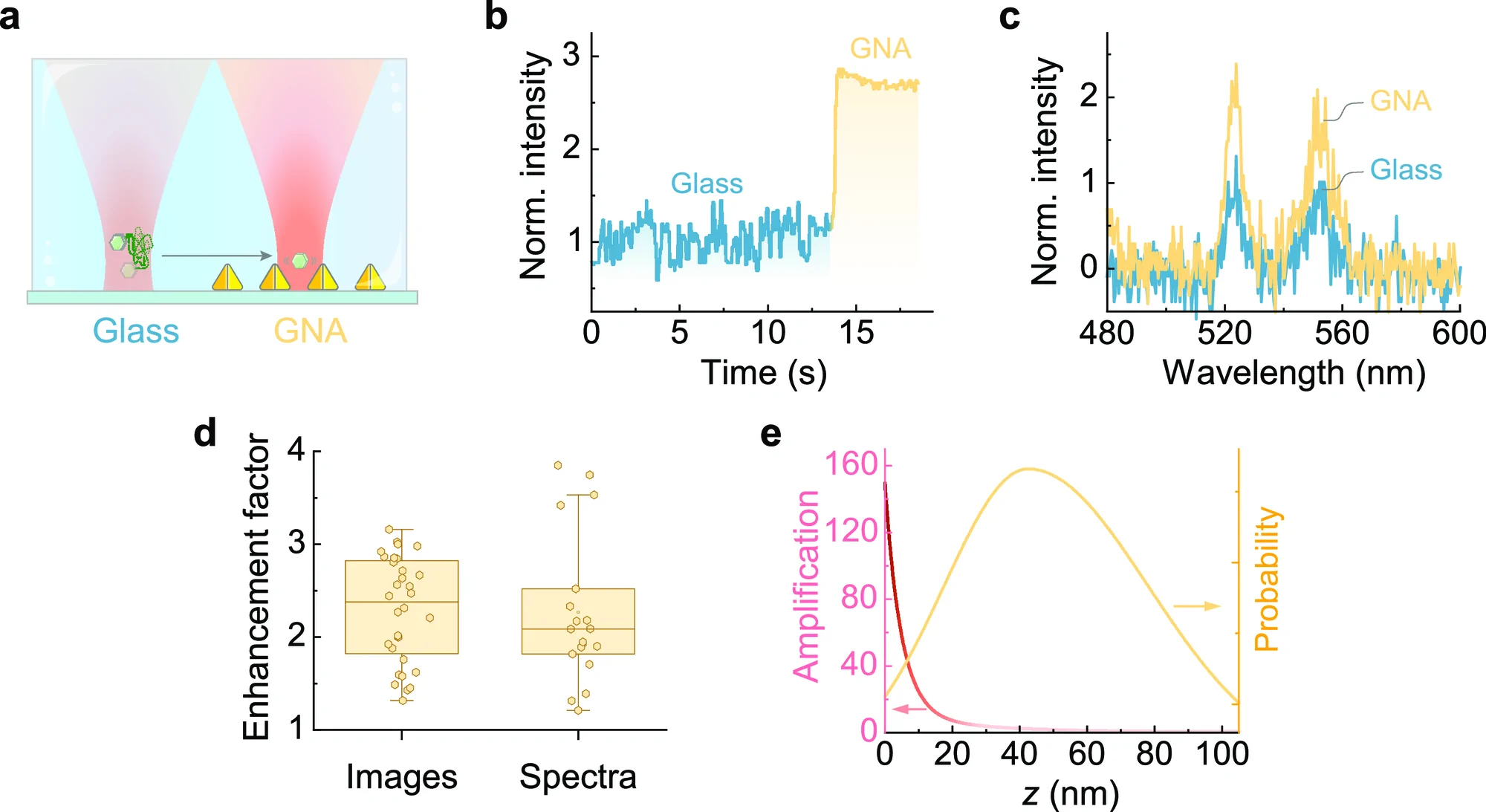
(a) Schematic diagram of a single UCNP driven from glass to the GNA-covered substrate for real-time observation of the plasmon-induced luminescence enhancement. (b) Time evolution of the luminescence intensity generated by an optically trapped UCNP during its displacement from the glass to the GNA-covered substrate. (c) Emission spectra corresponding to a UCNP when optically trapped on the bare glass and on the GNA-covered substrate. (d) Distribution of enhancement factors obtained from image sequences and emission spectra acquired during independent trapping experiments performed on different UCNPs. Each symbol represents an individual trapping event, while the box plots indicate the median, interquartile range, and overall data range. The mean enhancement factor obtained from both images and spectra is 2.3, with *n*_images_ = 32 and *n*_spectra_ = 19. *n* is the number of measurements. (e) Simulated 980 nm excitation intensity amplification factor as a function of the *z* (vertical) distance from the vertex of the gold nanopyramid, based on the numerical simulations presented in Figure 1d. The experimentally determined axial-position distribution of the UCNP when trapped on the GNA-covered substrate is also included.

The comparison of the emission spectra recorded when the UCNP was trapped on a glass substrate and on the plasmonic nanoantenna reveals differences not only in overall emission intensity but also in the spectral profile. These differences become more evident when the two bands constituting the green emission of erbium ions are fitted to Gaussian peaks (Figure 5a). Comparison of the actual emission spectra and the fitted curves corresponding to a single UCNP on glass and on the GNA array (Figure 5a) reveals how the intensity ratio *R* = *I*_H_/*I*_S_ increases when the UCNP is trapped near the gold nanoantenna, indicating an increase in its local temperature. For each excitation power, we determine the value of *R* for >10 independent trapping events. The dispersion in the values of *R* obtained is included in Figure 5b, as obtained for the three laser powers used in this work. These experimental data were used to calculate the corresponding average temperature increase for each laser power by using the calibration curve (Figure 5c). The error bars shown in Figure 5c correspond to the standard error of the mean obtained from the independent trapping experiments included in Figure 5b. The extracted temperature increases with excitation power and, within experimental uncertainty, follows an approximately linear trend. At the highest trapping power used in this study (60 mW, 11 MW cm^–2^), the UCNP luminescence reveals a temperature increase of approximately 40 °C. The uncertainty in temperature determination may originate from power-dependent calibration. This source of uncertainty is discussed in Section S3 of the Supporting Information. The power-dependent temperature increase corresponds to a heating rate of 0.6 °C mW^–1^, a value that aligns with those previously reported for similar plasmonic systems under comparable irradiation conditions (see Table S1 in the Supporting Information).^43–48^ Numerical simulations were performed to evaluate the steady state photothermal temperature distribution generated by the GNA as a function of absorbed powers at 980 nm. Different scenarios considering different locations of the 980 nm laser beam with respect to the GNA array are considered (Section S11 in the Supporting Information). The following discussion corresponds to the case in which the 980 nm laser is assumed to be focused on top of a GNA. From the resulting temperature maps, we extracted temperature profiles along the axial direction *z* at a lateral distance of 50 nm from the nanoantenna, selected to match the approximate distance derived from the experimental *x–y* position variance (Figure 3f). Comparison between the simulated temperature profiles and the experimental data indicates that the observed temperature rise is consistent with an absorbed power by the central GNA and by the GNA array of 2.7 × 10^–2^ mW and 6.7 × 10^–2^ mW, respectively. This indicates that only 0.1% of the total incident 980 nm excitation power is absorbed by the GNA array (more details about these calculations can be found in Section S11 of the Supporting Information). Although this absorbed fraction is lower than values commonly reported in the literature, it should be noted that the excitation wavelength used here is far from the plasmonic resonance, resulting in a reduced absorption cross-section for the nanoantenna. It should be emphasized that the experimentally determined temperature corresponds to the local temperature experienced by the UCNP at its trapping position. The temperature at the gold–water interface is not measured directly, but it is inferred by matching the experimental temperature increase to the simulated heat-distribution profiles. Once we have determined the 980 nm absorbed power by a single gold nanopyramid, it is possible to simulate the temperature map in its surroundings (see more details in Section S11 in the Supporting Information). Results presented in Figure 5d reveal that for an absorbed power (by the central pyramid) of 2.7 × 10^–2^ mW, the simulation estimates a temperature increase of approximately 60 °C at the gold–water interface (Figure 5e). This temperature increase at the nanoantenna is not expected to induce significant thermally driven reshaping of the gold nanostructures. Thermal annealing experiments presented in Section S12 of the Supporting Information show that the nanoantenna morphology remains largely preserved up to temperatures well above those estimated under our optical trapping conditions. Moreover, numerical simulations indicate that even a 10% reduction in nanoantenna size would not significantly modify the near-field enhancement at 980 nm, confirming that the optical performance of the GNA is retained. Further increasing the excitation power would be expected to substantially increase the local temperature at the gold–water interface, potentially promoting microbubble formation and compromising the stability of the optical trap.^49^

**Figure 5:**
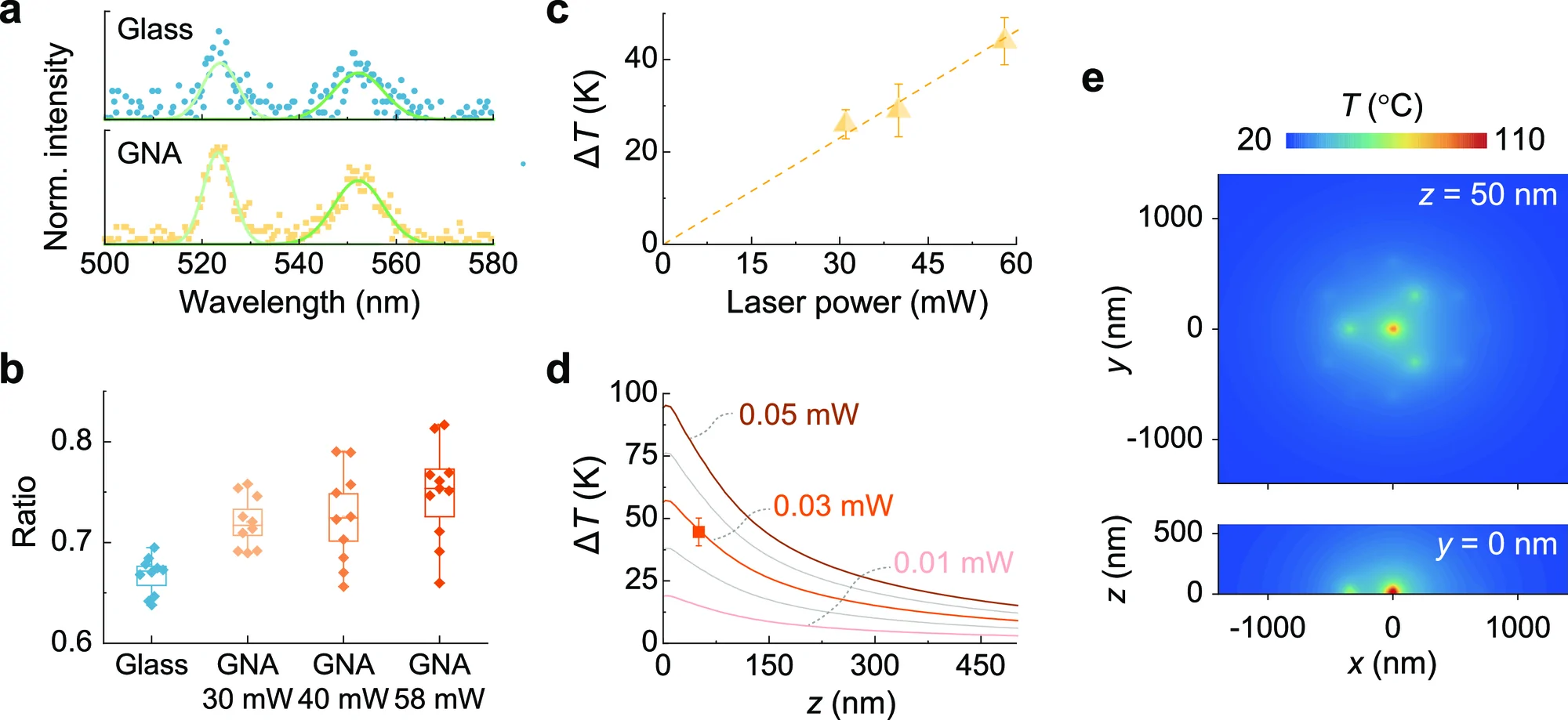
(a) Representative emission spectra of a single Er^3+^-doped UCNP recorded on a glass substrate (top) and in the vicinity of a gold nanoantenna (bottom). Symbols represent experimental data, while solid lines correspond to Gaussian fits used to extract the integrated intensities of the two Er^3+^ emission bands and calculate the intensity ratio, *R*. (b) Distribution of *R* obtained from independent trapping experiments performed on different UCNPs. Each symbol represents an individual trapping event, while the box plots indicate the median, interquartile range, and overall data range. The number of measurements performed on glass and on GNA at laser powers of 30, 40, and 58 mW was 10 for each condition. (c) Average temperature increase, Δ*T*, as a function of excitation laser power, calculated from the mean of *R* values obtained at each power. Error bars represent the standard error of the mean derived from the independent trapping experiments shown in (b). (d) Simulated axial temperature profiles obtained for different values of the 980 nm power absorbed by the central illuminated gold nanoantenna. Symbols indicate the experimentally determined temperature increase for a UCNP located approximately 50 nm above the substrate. (e) Top-view and cross-sectional temperature maps obtained from numerical simulations assuming a Gaussian beam focused on a gold nanoantenna and an absorbed power of 0.03 mW in the central nanoantenna. The top view in the (*x–y*) plane corresponds to an axial distance of 50 nm above the substrate.

From the temperature profile (Figure 5d), it is evident that the UCNP is optically trapped within a region exhibiting a non-negligible thermal gradient. Differentiation of the axial temperature distribution reveals that the particle experiences a gradient of d*T*/d*z* = −0.23 K nm^–1^ at *z* = 50 nm (Section S13 in the Supporting Information), where the negative sign reflects the monotonic decrease in temperature with increasing distance from the substrate. Such a thermal gradient gives rise to the appearance of thermophoretic force, which tends to drive the particle toward a cooler region, i.e., away from the gold nanopyramid. The effective thermophoretic force was estimated from the simulated temperature-gradient profile according to *F*_Th_ = –*k*_*B*_·*T*·*S*·∇*T*, where *k*_B_ is the Boltzmann constant, *T* is the local temperature, *S* is the Soret coefficient, and ∇*T* is the temperature gradient.^50^ Since the Soret coefficient of the UCNPs is not known, we used *S* = 0.1 K^–1^ as a conservative upper-bound value for dielectric nanoparticles in aqueous media. Under these conditions, the maximum effective thermophoretic force is estimated to be approximately 0.13 pN at *z* = 24 nm, decreasing to approximately 0.1 pN at the experimentally observed trapping position, *z* ≈50 nm (Section S14 in the Supporting Information). This value is smaller than the estimated optical restoring force, *F*_opt,max_ ≈ *k*_*z*_Δ*z*_*max*_ ≈0.45 pN, obtained using the effective axial trap stiffness *k*_*z*_ ∼ 9 pN μm^–1^ and the measured axial fluctuations. These estimates indicate that thermophoresis is not negligible and contributes to shifting the equilibrium position away from the nanoantenna surface, but that the optical restoring force remains dominant under our experimental conditions. The stable optical trapping observed throughout the experiments provides direct experimental evidence that thermophoretic forces do not dominate the particle dynamics. Indeed, in photothermal trapping systems, strong thermal effects have been reported to destabilize or even expel particles from the optical trap,^51^ a behavior not observed in our experiments. Additional contributions, including UCNP–substrate electrostatic interactions, are discussed in Section S14 of the Supporting Information.

In close proximity to a plasmonic nanostructure, several works report on the creation of convection currents.^52,53^ In our case, such currents could also affect the stability of the optical trap. The relevant parameter for buoyancy-driven convection is the Rayleigh number: ( $R_{a}=\frac{g\cdot \beta \cdot \Delta T\cdot H^{3}}{\nu \cdot \alpha }$ ) where *g* is gravity, β (≈2 × 10^–4^ K^–1^) is the thermal expansion coefficient of water, Δ*T* is the temperature increase caused by the heating source (200 K), *H* is the chamber height (120 μm), ν (≈10^–6^ m^2^ s^–1^) is the kinematic viscosity of water, and α (1.4 × 10^–7^ m^2^ s^–1^) is the water thermal diffusivity. Using these values, we obtain *R*_a_ ≈ 3.4. This value is orders of magnitude below the critical Rayleigh number required for the onset of natural convection in a fluid layer (≈1708).^54^ Therefore, buoyancy-driven convection is not expected to develop under our experimental conditions. In addition to these numbers, experimentally we do not observe signatures of convection, such as directional drift, long-range particle transport, or collective flow-induced motion. The observed dynamics are instead consistent with confined Brownian motion modified by optical and thermophoretic forces.

It is important to emphasize that the conclusions achieved in the previous paragraph, namely, the estimated nondominant role of effective thermophoresis forces and the absence of convective currents, apply specifically to our experimental conditions, in which the excitation wavelength is far detuned from the LSPR of the plasmonic nanostructure, resulting in moderate thermal gradients. Under resonant excitation, substantially stronger heating is expected, and the resulting thermophoretic forces and conductive currents could affect trap stability. Moreover, the use of larger optically trapped nanoparticles would further increase the Soret coefficient, thereby amplifying the thermophoretic forces. Under these conditions, photothermal effects could again approach or exceed the magnitude of optical forces, demanding a strategic approach to the design of optical trapping schemes near plasmonic nanostructures.

## Conclusions

In summary, this work demonstrates the capability of individual upconverting nanoparticles to perform thermal measurements in the immediate vicinity of plasmonic nanoantennas. Through the integration of single-beam optical trapping, luminescence imaging, and spectral analysis, we achieved simultaneous determination of both the three-dimensional position distribution and the average temperature of a single UCNP positioned near a gold pyramid-like nanoantenna. Analysis of position histograms, derived from luminescence images of a single UCNP, reveals that local enhancement of the 980 nm excitation and trapping radiation results in both improved spatial confinement and increased emission intensity. This enhancement is directly related to the plasmonic resonance occurring at the vertices of the nanoantenna, which is shown to produce more than a 2-fold increase in the luminescence from an individual UCNP. Spectroscopic analysis indicates that plasmonic heating near (50 nm away, where the UCNP is trapped) the nanoantenna yields a heating rate of 0.6 °C mW^–1^. Under the experimental conditions employed, this effect leads to single-particle temperature increments approaching 40 °C. The combination of these experimental data with computational simulations suggests the presence of temperature increments at the gold–water interface close to 100 °C, with prospects for even higher localized temperatures at increased excitation powers. Despite the significant heating, model-based estimates indicated that the resulting thermophoretic forces are substantially weaker than the optical trapping forces. Specifically, the thermophoretic force is estimated to be approximately 15 fN, whereas a trap stiffness is approximately 9 pN·μm^–1^. These estimated force magnitudes are consistent with the observed stable confinement of the UCNP near the nanoantenna under the conditions studied.

The experimental results here shed light on the interplay of optical and thermal phenomena at the nanoscale and offer important guidance for designing trapping and sensing platforms that operate near plasmonic nanostructures.

## Methods

### Excitation Power-Dependent Emission Measurement

The UCNP samples to be measured were prepared as follows. A spacer with a diameter of 20 mm and a height of 0.12 mm (Secure-Seal) was fixed onto a glass slide. A dilute aqueous UCNP suspension (identical to that used for optical trapping experiments) was then drop-cast within the area defined by the spacer, followed by solvent evaporation under ambient conditions. 20 μL of distilled water was added into the spacer, and the immobilized UCNPs at the bottom, along with water, were sealed by placing a glass coverslip on top. The laser beam was focused through an oil immersion objective onto the surface of the glass slide to identify small UCNP aggregates. The excitation power was increased step by step, with a corresponding luminescence image recorded at each intensity level.

### Analysis of the Luminescence Intensity

The luminescence intensity generated by a single UCNP was characterized via luminescence imaging and spectroscopy using the integrated optical trapping system (Figure S3). The luminescence intensity from image sequences was analyzed by using ImageJ software (National Institutes of Health, USA).^55^ The bright spot was selected by a circular region of interest, and the intensity of the highest-value pixel within the region was utilized as the representative luminescence intensity. The images were not subjected to any modification, except that the background was subtracted prior to analysis. Spectral intensity was determined by calculating the integrated signal of the emission bands within the 520–560 nm range.

### Temperature Calibration Based on the Luminescence Intensity Ratio

The sample used in this experiment was identical to that used in the excitation power-dependent emission measurement. To investigate the evolution of the spectrum with temperature, a heating plate (LINKAM PE120, United Kingdom) was placed under the glass slide to control the temperature of the UCNPs. The emission spectra were collected via the spectroscopic detection path of the optical trapping setup (Figure S3) in the 20–83 °C temperature range. The integration time for each spectral acquisition was set to 10 s. The *I*_H_ and *I*_S_ that were used for the intensity ratio calculation (*R* = *I*_H_/*I*_S_) are the integrated intensities over the 520–535 nm and 535–560 nm spectral bands, respectively.

### Three-Dimensional Single UCNP Tracking

A particle-tracking approach based on luminescence imaging was employed to construct the three-dimensional trajectories of the optically trapped UCNPs. The lateral coordinates (*x*, *y*) were extracted using a computational algorithm, SpotTrack, which is optimized for finding the time-trajectory of a single particle in highly noisy image sequences.^56^ The axial position *z* was determined by exploiting the correlation between the particle’s displacement from the focal plane and the concomitant broadening of its luminescence spot. The vectorial Debye diffraction integral theory was applied to model the tightly focused optical fields in high-NA systems. By decomposing the incident wavefront, the field distribution near the focus was expressed as a superposition of plane waves with wave vectors converging toward the focal point. The numerical simulations of *z* positioning, obtained using the Debye vector diffraction integral method, are presented in Figure S6. The simulation assumed an NA of 1.4, a refractive index of 1.51, and a focal depth of 125 μm below the interface, under an index-matched condition. To simplify the computational process, the incident wavelength was modeled using eight equally spaced plane waves ranging from 620 to 680 nm. The final optical field was obtained by calculating the field for each wavelength individually and then superimposing the results.

### Numerical Simulation of Electric Field Distribution

Nanoantennas were modeled as rounded tetrahedra of the size extracted from the SEM analysis and simulated using commercial FDTD software (Lumerical, Ansys). The gold refractive index was given by D. Yakubovsky et al.,^57^ while glass and water refractive indices were fixed to 1.51 and 1.33, respectively. First, plane wave simulations were carried out for analyzing the spectral features of the periodic structure. To further understand the trapping mechanism, the calculations based on a Gaussian beam using a 5 μm × 5 μm simulation domain without periodic boundary conditions were performed. The 1 μm diameter laser spot was focused on three different regions of the array: (i) the center of six nanoantennas, (ii) the top of a nanoantenna, and (iii) the gap between two adjacent nanoantennas (Figure S9).

## Supplementary Material









## Notes

Fabrication of gold nanoantenna array; synthesis of upconverting nanoparticles; excitation-power dependence of thermometric calibration; single particle experiments; optical trapping setup; trapping in a multisite plasmonic potential; optical trap stiffness calculation; *z*-axial localization of the UCNP; simulations of the electric field enhancement; previously reported measurement of plasmonic heating; simulations of the temperature map; thermal stability of gold nanoantenna; thermal gradient and effective thermophoretic force; and optical, electrostatic, and effective thermophoretic potentials (PDF)
*E* ^2^ intensity evolution with wavelength (AVI)
Scanning the UCNP from GNA to glass (AVI)
Discrete jump of UCNP being scanned on GNA (AVI)

## Abbreviations

LSPR: localized surface plasmon resonance
UCNP: upconverting nanoparticle
GNA: gold nanoantenna array
SEM: scanning electron microscopy
TEM: transmission electron microscopy
FWHM: full width at half-maximum
