# Supplementary material for: Single Particle Nanothermometry Quantifies Heating in a Plasmonic Trap
Source: ACS Nano. 2026 Aug 13;20(33):23223–33. doi: 10.1021/acsnano.6c03979 (PMC13523793; doi:10.1021/acsnano.6c03979)
Supplement: Supplementary file 1 [file nn6c03979_si_006.pdf]

Supporting Information for

Single Particle Nanothermometry Quantifies Heating in a Plasmonic Trap

Fengchan Zhang,[1,2] Manuel Romero,[3] Khouloud Hamraoui,[4] Jiachen Zheng,[5] Thi Tuyen Ngo,[3] Ginés Lifante-Pedrola,[2] Gabriel Lozano,[3] Hernán Míguez,[3] Fan Wang,[5] Jorge Rubio-Retama,[4] Daniel Jaque,[1,2,] Patricia Haro González[1,2]*

[1]Nanomaterials for Bioimaging Group (nanoBIG), Departamento de Física de Materiales, Facultad de Ciencias, Universidad Autónoma de Madrid, 28049 Madrid, Spain
[2]Instituto Nicolás Cabrera (INC), Facultad de Ciencias, Universidad Autónoma de Madrid, 28049 Madrid, Spain
[3]Institute of Materials Science of Seville, Spanish National Research Council, University of Seville, 41092 Seville, Spain
[4]MatNaBIO Research Group, Department of Chemistry in Pharmaceutical Sciences, Faculty of Pharmacy, Complutense University of Madrid, 28040 Madrid, Spain
[5]School of Physics, Beihang University, Beijing, People's Republic of China
*daniel.jaque@uam.es

Section S1. Fabrication of gold nanoantenna array

The gold nanoantenna array was fabricated using a wedge evaporation method.[1] An ordered 720 nm polystyrene (PS) sphere monolayer was first deposited on a glass substrate using an aqueous suspension with a concentration of 2.1% vol.[2] The substrate was inclined at a 3° angle, and 300 μL of the suspension was left for 2 days at temperature ranging from 20 and 30 °C and at relative humidity of 90% for water evaporation. Then, a 100 nm thick gold layer was deposited via thermal evaporation using a Univex 250 vacuum coating system (Leybold, Germany). Finally, the PS spheres were removed by sonication in absolute ethanol for 2 min. A periodic array of gold nanopyramids arranged in a hexagonal lattice was created.

Section S2. Synthesis of upconverting nanoparticles

Materials

Yttrium (III) chloride hexahydrate (YCl3·6H2O, 99.99%), ytterbium (III) chloride hexahydrate ($YbCl_3·6H_2O$, 99.9%), erbium (III) chloride hexahydrate ($ErCl_3·6H_2O$, 99.9%), 1-octadecene (ODE, 90%), oleic acid (OA, 90%), sodium hydroxide (98%), ammonium fluoride (98%), methanol (99.9%), N,N-dimethylformamide (DMF, 99%), Nitrosyl tetrafluoroborate ($NOBF_4$, 95%), ethanol absolute (EtOH), n-hexane (97%), acetone (99%), toluene (⩾99.5%) were purchased from Sigma-Aldrich (Merck). Poly (acrylic acid) (PAA), 50 wt.% solution in water; approx. MW 3000 to 5000 was purchased from Thermo Fisher Scientific.

Synthesis

The synthesis of $NaYF_4$: 20% Yb^{3+},2% Er^{3+} UCNPs followed a thermal co-precipitation method.[3] $ErCl_3·6H_2O$ (7.63 mg, 0.002 mmol), $YbCl_3·6H_2O$ (77.49 mg, 0.2 mmol), and $YCl_3·6H_2O$ (236.54 mg, 0.78 mmol) were dissolved in a mixture of 15 mL 1-octadecene and 6 mL oleic acid in a three-neck round-bottom flask equipped with a reflux condenser, a thermocouple for continuous temperature monitoring, and connections to vacuum and inert gas (N_2) lines. Note that the probe was not independently calibrated against a NIST-traceable or accredited standard within the preceding year. Nevertheless, the observed reaction conditions were consistent with expected values for this system, and the synthesis showed good batch-to-batch reproducibility, suggesting that this limitation does not significantly impact the experimental outcome. The solution was heated to 140 °C under a nitrogen atmosphere using a temperature-controlled heating mantle. The temperature was precisely regulated by an ELECTEMP-TFT digital temperature controller (J.P. Selecta S.A., Barcelona, Spain). Once the target temperature was reached, the system was switched to vacuum, maintained at 140 °C for 20 min and then cooled to room temperature. Vacuum was provided by a Telstar vacuum pump (Terrassa, Spain), and the system was evacuated to approximately 100 mbar prior to the reaction. Separately, NaOH (100 mg, 2.5 mmol) and NH_4F (148 mg, 4 mmol) were dissolved in 10 mL of methanol and added dropwise to the rare-earth solution under vigorous stirring at room temperature. After stirring for 30 min, the mixture was heated to 110 °C under nitrogen and maintained under vacuum for 20 min to remove methanol, then further heated to 315 °C under nitrogen using a reflux setup and maintained for 1 h to promote nanocrystal growth. After the reaction, the mixture was cooled to room temperature. The product was precipitated with methanol, vortexed, and the phases were allowed to separate. The methanol phase was discarded, and this separation process was performed twice. The remaining organic phase was centrifuged (8500 rpm, 20 min) using a centrifuge from J.P. Selecta S.A. (Barcelona, Spain), and the collected nanoparticles were washed twice with absolute ethanol (8500 rpm, 20 min). Finally, the oleate-capped UCNPs were redispersed in 5 mL of hexane and stored for further use.

To prepare the obtained UCNPs for optical trapping experiment in distilled water, a ligand-exchange procedure was used. Briefly, 5mL N,N-dimethylformamide (DMF) was added to 2 mL oleate-capped UCNPs in hexane (~6 mg mL^{-1}), and the mixture was stirred at room temperature for 5 min. Subsequently, nitrosyl tetrafluoroborate ($NOBF_4$, 150 mg, 1.28 mmol) was added, and the mixture was stirred at room temperature for additional 30 min for ligand strip. To purify the UCNPs, 5 mL toluene and 5 mL hexane were added to flocculate the UCNPs dispersion. The mixture was then centrifuged at 11000 rpm for 30 min. After discarding the supernatant, the precipitated UCNPs were re-dispered in 2 mL DMF with the aid of sonication for 2 min to form a stable colloidal dispersion. To perform the secondary ligand-exchange reaction, a solution of poly(acrylic acid) (PAA, 500 mg, 6.9 mmol) in 2 mL DMF was prepared by heating and stirring at 80 °C for 10 min. Then the UCNPs/DMF dispersion was added drop by drop to the aforementioned PAA solution and then heated and stirred at 80 °C for 30 min. After centrifugation at 11000 rmp for 30 min, the supernatant was removed. The precipitation of UCNPs was washed with acetone twice, with distilled water twice, and redispersed in water for future use.

Section S3. Excitation-power dependence of the UCNP thermometric calibration

The excitation densities used in this work are in the MW cm^{-2} range, which is the regime required for optical trapping. Previous studies have shown that the reduction of thermal sensitivity occurs once the excitation density exceeds a saturation threshold, due to the increasing role of non-Boltzmann population dynamics and multiphonon relaxation.[4] Importantly, after this threshold is reached, the relative thermal sensitivity becomes much less dependent on further increases in excitation density. Therefore, even though the plasmonic nanoantenna locally enhances the excitation field, the UCNPs are already operating in the saturated high-power regime even in the absence of the nanoantenna. For this reason, we do not expect the plasmonic enhancement to introduce a major additional change in the thermal sensitivity. To support this, we measured the calibration curves in a wide range of excitation power intensities (4-23 MW/cm^2). Results are shown in **Figure S1**. The normalized relative thermal sensitivities calculated from the calibration data in **Figure S1a** are included in **Figure S1b**. In accordance with L.Marciniak and co-workers[4] a reduction in the thermal sensitivity as the pump power intensity. Increases are observed. Our experimental data reveal that the 980 nm pump intensity is expected to increase by 4 times when moving from glass to gold nanoantenna. For this increment in the power intensity, data included in **Figure S1b** reveals a change in the relative sensitivity close to 10%. The uncertainty that such reduction would induce in the thermal readout ($T = T_0 + \Delta T = T_0 + \frac{1}{S_r}\frac{\Delta R}{R}$) would be given by:

$$\delta T = \Delta T \frac{\delta S_r}{S_r} \qquad \textbf{[Eq. S1]}$$

We, therefore, estimate that the change in the thermal sensitivity that could happen in the plasmonic trap introduces an uncertainty in the thermal readout of 4 ºC.

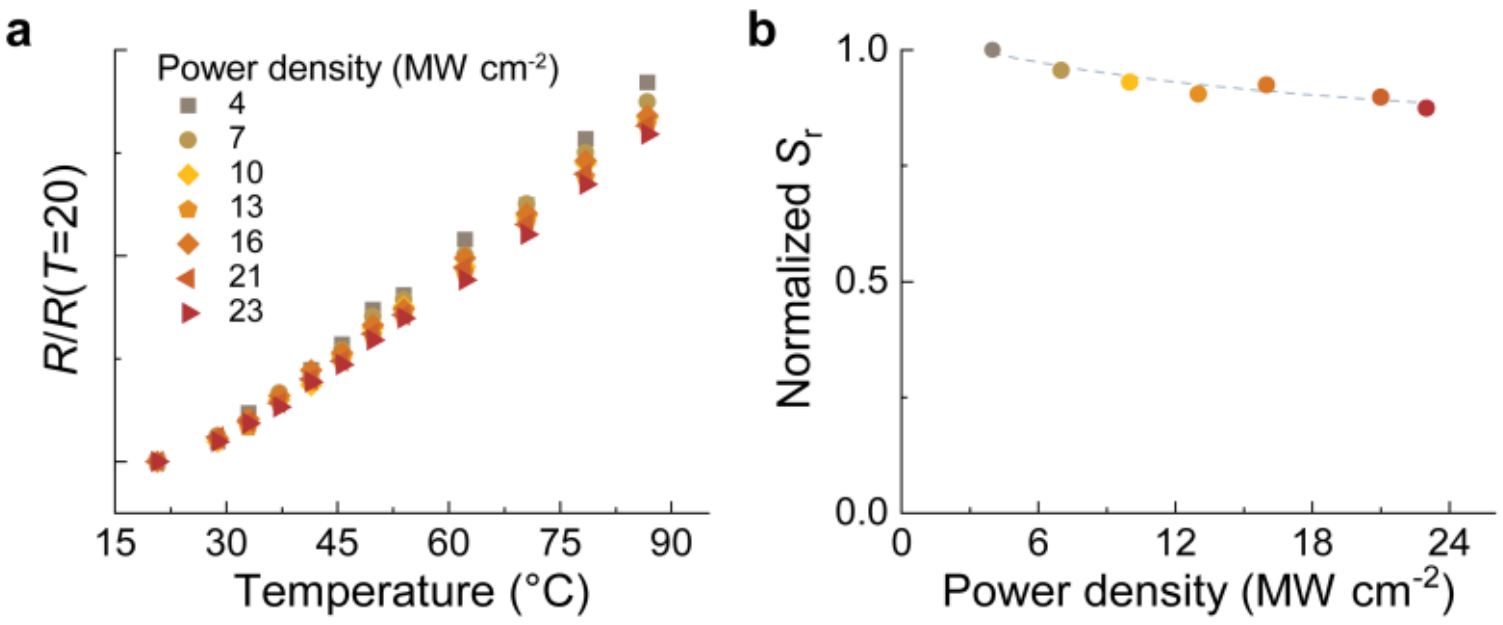

Figure S1. (a) Normalized thermometric parameter $R/R(T{=}20$ ºC) as a function of temperature for different 980 nm excitation power densities ranging from 4 to 23 MW cm^{-2}. **(b)** Relative thermal sensitivity, S_r normalized to its value at 4 MW cm^{-2}, as a function of excitation power density. Dots are experimental data and dashed line is a guide for the eyes.

At the excitation power densities used in this work (MW cm^{-2}), direct multiphoton excitation and saturation processes can contribute significantly to the populations of the Er^{3+} $^2H_{11/2}$ and $^4S_{3/2}$ levels, so that strict Boltzmann equilibrium between these states cannot be assumed. Consequently, the luminescence intensity ratio should not be interpreted as a purely thermodynamic Boltzmann parameter. Instead, the temperature is obtained from an empirical calibration performed under the same high-power excitation conditions used in the trapping experiments, which inherently incorporates these non-equilibrium population dynamics. The resulting temperatures should therefore be regarded as effective temperatures within this calibration framework. A systematic bias may arise if the excitation conditions during measurement differ substantially from those used for calibration but in our conditions (where similar power intensities were used) such bias is not expected to occur.

Section S4. Evidence of single particle experiments

In our work we deal with a diffraction limited spot whose volume is much larger than the volume of a single UCNP. If we estimate the volume of the trap as $V_l = w_0^2 \cdot l_R$ = 2.5·10^8 nm^3 where w_0^2 and l_R are the beam waist and Rayleigh distance of the 980 nm excitation beam (500 and 1000 nm, respectively) we conclude that up to 10^4 UCNPs could be simultaneously trapped (the volume of a single UCNP is estimated to be 1.12·10^4 nm^3). In our work we adopted different strategies to ensure that experimental data do correspond to single UCNP trapping events:

i) We work with highly diluted solutions (< 10^{-7} mg/mL) that lead to UCNP-UCNP distances larger than 100 microns. In these conditions the time required for a UCNP to reach the trap can be estimated as $t = \frac{d^2}{6D}$ = 6 mins, where $D = \frac{k_B T}{6\pi\eta R}$ is the diffusion coefficient for a UCNP in water where η is water viscosity and R is the radius of the UCNP. We can assume that this is the time between multiple trapping events that is larger than the duration of our experiments. Therefore, we conclude that during acquisition of experimental data the probability of trapping more than one UCNP is very limited. We should clarify that we start the experiments once the UCNP is trapped and that typically the process of trapping this first UCNP takes always more than 5 mins.

ii) During trapping experiments the incorporation of secondary UCNPs can be easily detected by monitoring the intensity vs time curve. Each time that an additional UCNP incorporates into the trap, this is evidenced by the appearance in a step like intensity increment in the detected intensity. A typical intensity vs time curve showing the evidence of event is shown in **Figure S2**. Note that the incorporation of any additional UCNP would lead to a sharp increase in the collected intensity. When this happens during experiments, we re-start them and data are not included in our calculations.

iii) In our experiments we perform cyclic experiments driving the UCNP from the glass to the nanoantenna and vice-versa, so we can compare the intensity at the glass before and after testing the plasmonic enhancement. In all cases the intensity was found similar, without evidence of the incorporation of an additional UCNP and ensuring that results correspond to a single particle (**Figure S2**).

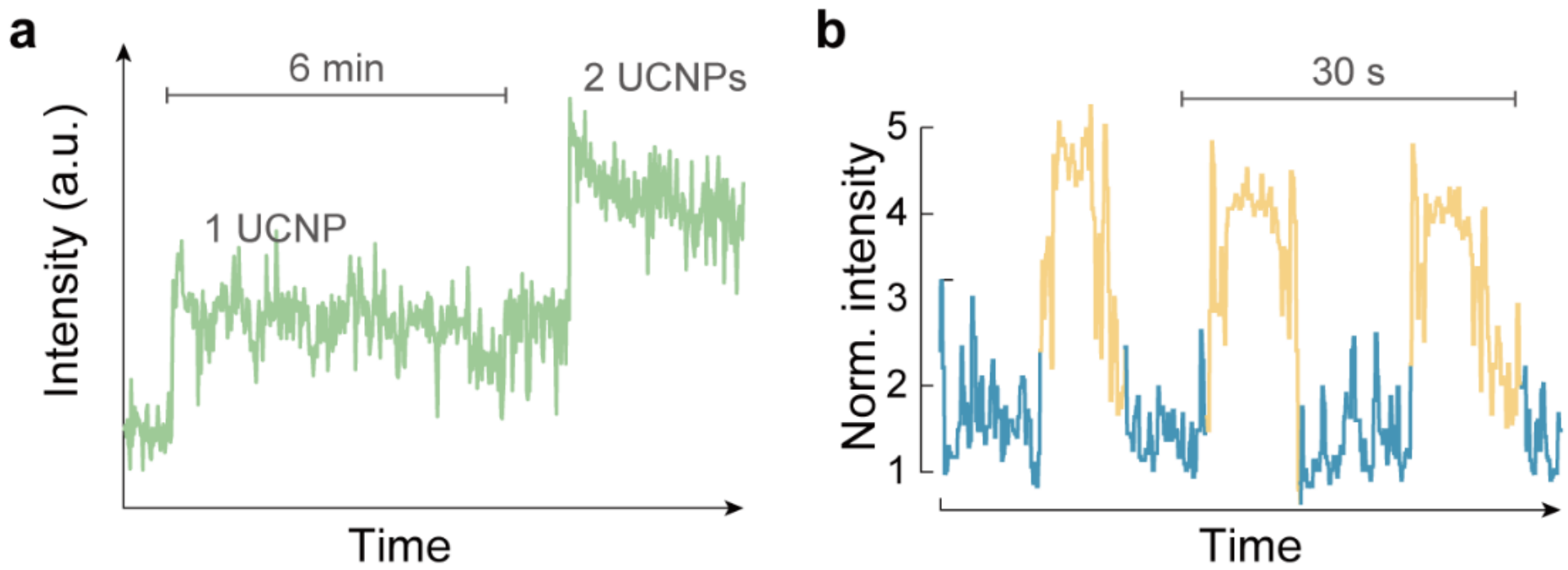

Figure S2. (a) The time evolution of luminescence intensity generated by optically trapped UCNPs. The step-like intensity increase indicates the sequential incorporation of an extra UCNP into the trap. **(b)** The luminescence intensity variation of a single trapped UCNP as it moves vice-versa between the glass substrate (blue) and the gold nanoantenna (yellow).

Section S5. Optical trapping setup

A home-built single-beam optical tweezers setup was used to manipulate a single UCNP, with integrated imaging and spectroscopic functions (**Figure S3**). The optical trapping beam was provided by a single-mode fiber-coupled continuous-wave laser diode operating at 980 nm (Lumics, Berlin, Germany, cat. no. SN0700548). The fiber output was collimated using a FiberPort collimator (PAF2-7B, Thorlabs, Newton, NJ, USA), specified for the 600–1050 nm spectral range and equipped with an aspheric lens of focal length (f = 7.5) mm. According to the manufacturer specifications, this collimator provides a nominal collimated beam diameter of approximately 1.62 mm at the exit of collimator for the corresponding wavelength range. The collimated 980 nm beam was directed into the above-mentioned 100× oil-immersion microscope objective with numerical aperture NA = 1.4 (UPLSAPO100XO/1.4, Olympus/Evident, Japan). The back aperture of the microscope objective (as provided by manufacturer) was approximately 5 mm. In our optical configuration, the laser beam diameter at the objective back aperture was estimated to be approximately 2 mm. Therefore, the objective back aperture was intentionally underfilled, with a filling fraction of $F_{ob} = \frac{D_{beam}}{D_{pupil}} \approx 0.4$. This corresponds to an effective numerical aperture of $NA_{eff} \approx F_{ob} \times NA \approx 0.56$. Assuming a diffraction-limited Gaussian focus, the beam waist at the sample plane can be estimated as $w_0 = \lambda_l/\pi \cdot NA_{eff}$, where λ_l is the trapping laser wavelength (980 nm). Therefore, the estimated beam waist at the sample plane was approximately $w_0 \approx 557\ nm$. The corresponding Rayleigh range was estimated as $z_R \approx \pi \times w_0{}^2 / \lambda_l \approx 1$ µm.

The choice of an underfilled-aperture configuration was based on our experimental experience with optical trapping of luminescent nanoparticles close to dielectric and plasmonic interfaces. Although filling the full objective back aperture would increase the effective numerical aperture and, in principle, the maximum attainable trap stiffness, it would also increase the contribution of high-angle marginal rays. These rays are more sensitive to spherical aberration, refractive-index mismatch at the oil/glass/water interfaces, small beam misalignments, and optical imperfections. In practice, we found that a moderately underfilled configuration provides a cleaner and more reproducible Gaussian excitation profile. This underfilled configuration also increases the axial extension of the focal volume, making the optical trap more tolerant to small axial displacements of the nanoparticle and to imperfect positioning with respect to the nanoantenna array. This point is particularly relevant in the present experiments, where the trapped UCNP is located close to a nanostructured plasmonic substrate and experiences Brownian motion, near-wall effects, local thermal gradients, and possible thermophoretic contributions. Stable optical trapping of single UCNPs was consistently achieved under these conditions, confirming that the effective gradient forces were sufficient for the experiments reported in this work.

The laser beam not only functioned as an optical trap for a single UCNP but also an excitation source for its luminescence. When an optical trapping experiment is carried out in the presence of the gold nanoantennas, the laser also served to excite the localized surface plasmons.

The optical trapping chamber was created by attaching 125 µm thick double-sided adhesive tape to both sides of the region covered with gold nanoantenna arrays, followed by covering the structure with a glass coverslip. The resulting chamber formed an open-ended rectangular channel with a height of 125 µm, sealed on two sides and exposed on two sides. The lateral dimensions of the chamber were not strictly controlled and were typically around 18 mm × 18 mm, with slight variations between different experiments (**Figure S4**). The dispersion of upconverting nanoparticles was then introduced into the microchamber using a micropipette. The trapping chamber was placed on a motorized translation stage (ZFS13B, Thorlabs and 144198, maxon motor, Switzerland) enabling movement in both the vertical and horizontal directions. When a single UCNP was optically trapped, its luminescence was collected

by using the oil immersion microscope objective on top. After reaching a 50:50 beamsplitter (EBS1, Thorlabs), the luminescence signal was split into two. A portion of the signal was reflected by the beamsplitter and collected by a CCD camera (QIClick-F-M-12, QImaging, Surrey, British Columbia, Canada) to visualize the trapping event and quantify the luminescence intensity. Another portion of the signal passed through the beamsplitter, was reflected by a dichroic mirror (DMLP900, Thorlabs), and subsequently focused by a lens (LA1509-A, Thorlabs) into an optical fiber (QP400-2-SR, OceanOptics, USA) that coupled to a spectrometer (QE65000, OceanOptics, USA) for spectral analysis. In both cases a shortpass filter (FESH0750, Thorlabs) was used to eliminate the laser.

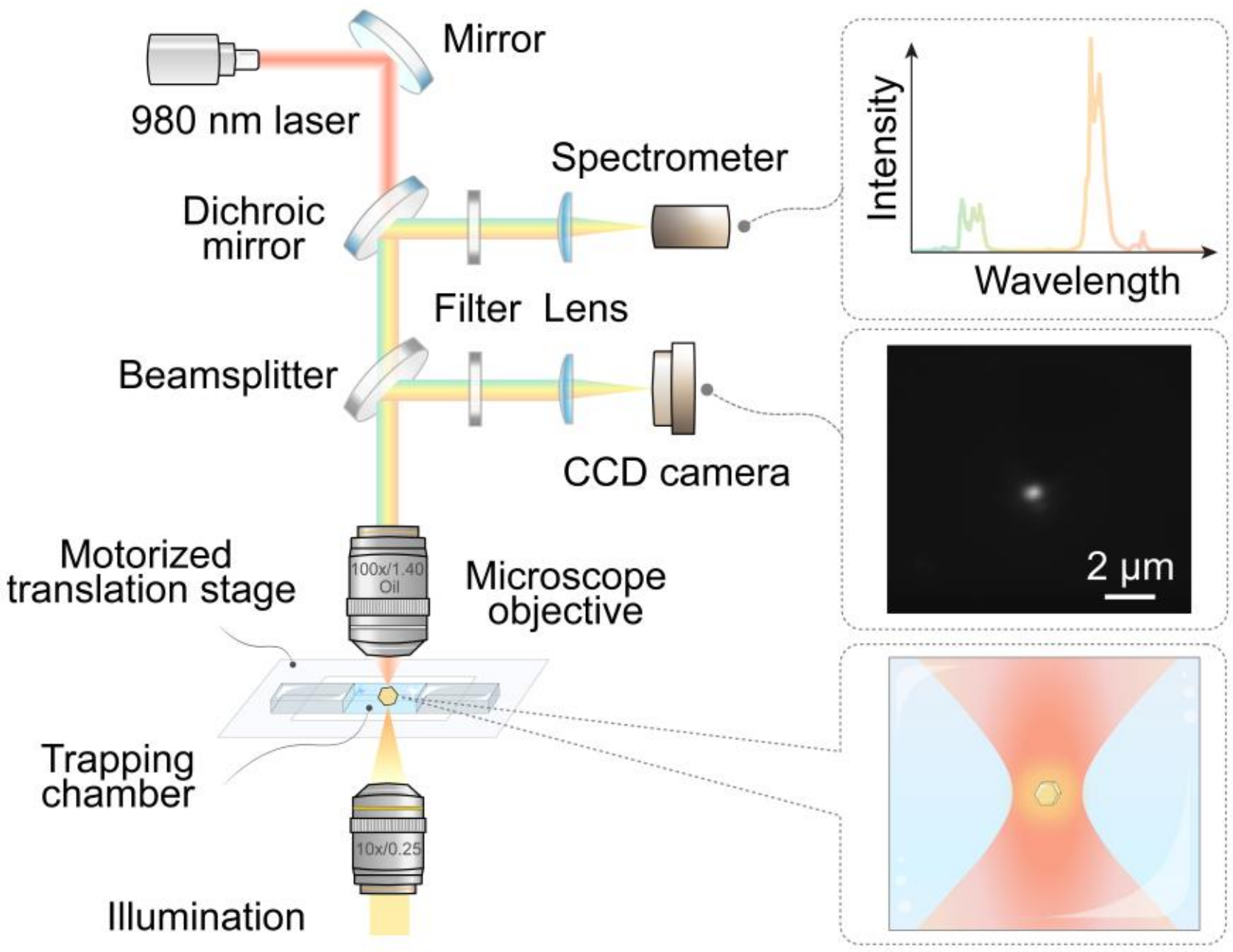

Figure S3. Schematic diagram of the optical setup for optical trapping and manipulation of a single nanoparticle, luminescence imaging, and spectral measurement.

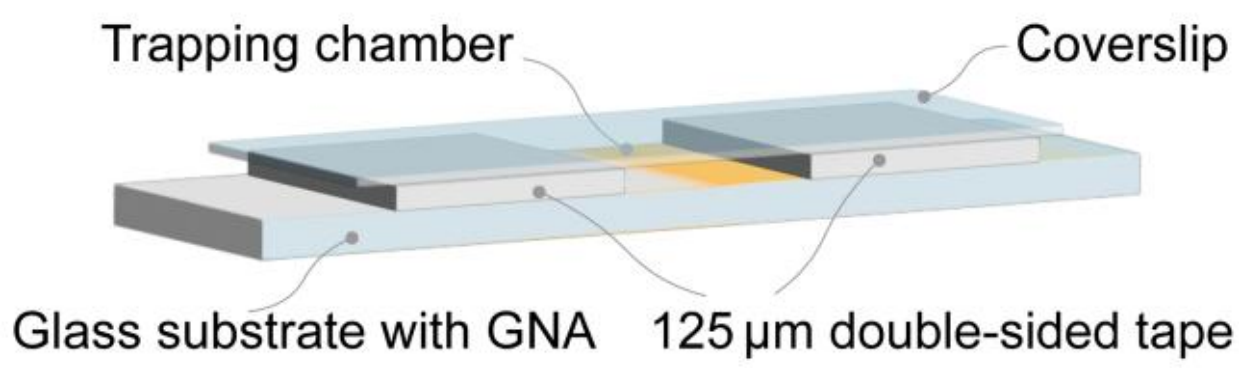

Figure S4. Schematic of the optical trapping chamber. Not drawn to scale.

Section S6. Single-UCNP trapping in a multi-site plasmonic potential

Given the ~1 µm excitation spot and the 190 nm spacing between nanoantennas, several nanopyramids are simultaneously illuminated, potentially generating multiple trapping hotspots. In principle, this could lead to simultaneous trapping of multiple UCNPs or aggregates if the particle concentration were sufficiently high. However, this situation does not apply in our experiments. The UCNP dispersion is highly diluted, with average interparticle distances on the order of ~100 µm, which is several orders of magnitude larger than both the inter-antenna spacing (~190 nm) and the excitation spot size (~1 µm). Under these conditions, the probability of having more than one UCNP within the trapping volume is negligible. In addition, all experiments are performed by first trapping a single UCNP on the bare glass substrate and then moving this single UCNP into the patterned substrate.

Once on the patterned substrate, multiple nanoantennas are illuminated simultaneously, creating several potential trapping sites. In static conditions, when the 980 nm trapping laser and the GNA are fixed, the UCNP is trapped at a fixed position that corresponds to the location where the largest plasmonic-induced optical gradient is produced (at the vertex of the nanoantenna illuminated with the largest 980 nm laser intensity). When we moved from static conditions and the 980 nm laser is scanned over the GNA array, the 980 nm laser intensity reaching the different GNA is changed and the UCNP "jumps" into the best illuminated GNA. In this situation (980 nm beam scanning over the GNA array), the UCNP can indeed explore different hotspots within the array. **Figure S5** and **Video S3** constitute experimental evidence on how the UCNP undergoes discrete changes in its equilibrium position (it jumps from one GNA to another) when the 980 nm beam is scanned over the GNA array. Note that no luminescence signal is observed at the position before the UCNP jumps into it, indicating that no particle was trapped there beforehand. This observation further supports that a single particle is being manipulated within a multi-site potential landscape, rather than multiple particles being trapped simultaneously. In the experiments included in the manuscript, the 980 nm laser beam and the GNA array remained in static condition, so all the experimental data included in the manuscript correspond to a single UCNP interacting with the same GNA.

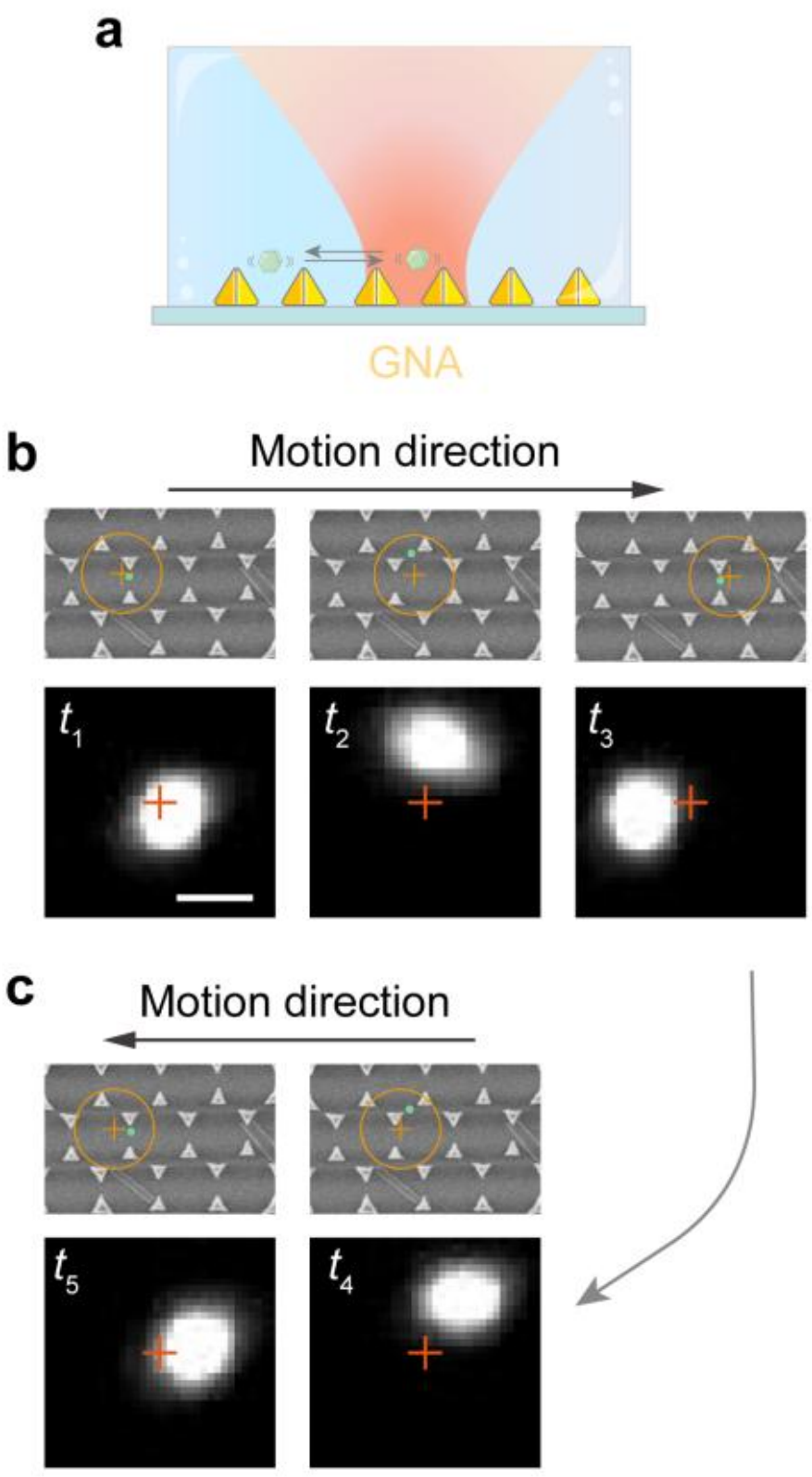

Figure S5. **(a)** Schematic of the back-and-forth horizontal scanning of an optically trapped UCNP over a GNA-covered substrate. **(b)** Three representative positions taken at t_1, t_2, and t_3 during the movement. During scanning, the relative position between the laser beam and the substrate changes, causing the nanoparticle to be trapped at the nanoantenna tip rather than at the laser focal point (see more details in Figure S9). **(c)** After t_3, the UCNP was driven back along the same path, returning to its previously trapped positions. The positions at t_4 and t_5 correspond to those at t_2 and t_1, respectively. The orange circle represents the laser beam, the orange cross represents the beam center, the green hexagon represents the UCNP. Scale bar: 1 μm.

Section S7. Optical trap stiffness calculation

The thermal fluctuations of a trapped particle provide an independent method to quantify the trap stiffness through application of the equipartition theorem.[5] For a particle confined in a harmonic potential with stiffness k_x, the equipartition theorem establishes the following relationship:

$$\frac{1}{2}k_B T = \frac{1}{2}k_x\left(x - x_{eq}\right)^2 \quad \textbf{[Eq. S3]}$$

where k_B is the is the Boltzmann constant, T is the absolute temperature, k_x is the trap stiffness along x-axis, x_{eq} is the equilibrium position of the trapped particle. Thus, the trap stiffness can be determined by measuring the positional variance of the particle, $k_x = k_B T/\left(x - x_{eq}\right)^2$. Under this method, prior knowledge of the particle shape or the viscosity of the surrounding medium is not required (although, in fairness, both the particle geometry and the optical properties of the medium can slightly influence the position calibration itself), but only the local temperature needs to be known.

When trapping on the GNA array, the UCNP dynamics are governed not only by restoring optical force, but also by the effective thermophoretic force and the repulsive interaction with the glass substrate forming the bottom of the trapping chamber (see **Section S14**). Consequently, the trap stiffness derived using the equipartition theorem represents an effective trap stiffness, as explicitly indicated in the main text.

Section S8. *z*-axial localization of the UCNP: procedure and uncertainty

Numerical simulations were used to estimate the axial position of the UCNP under the trapping conditions. The axial localization relies on the variation of the apparent size of the luminescence spot as the nanoparticle moves away from the focal plane. Briefly: using vectorial diffraction simulations under the optical conditions of our setup, we calculated the fluorescence PSF of a single emitter as a function of axial displacement.[6] This allowed us to construct a calibration curve relating the FWHM of the luminescence spot to the axial position (**Figure S6**). The experimentally measured FWHM values extracted from luminescence videos were then converted into *z* positions using this calibration.

The procedure is next described in more detail. First, we theoretically calculated the point spread function (PSF) of a particle at different axial positions. From each simulated PSF image, we extracted the full width at half maximum (FWHM) of the fluorescence spot, thereby establishing a calibration curve relating the FWHM to the axial distance from the substrate surface (**Figure S6a**). Because the focal plane was set at the substrate surface, the FWHM increased monotonically as the particle moved away from the surface. Therefore, each measured FWHM corresponded to a unique axial position, avoiding ambiguity in axial position determination. Second, during the experiment, the acquired images were analyzed frame by frame. For each frame, we extracted the experimental PSF of the particle and calculated the corresponding FWHM (**Figure S6b**). Then the axial position of the particle in that frame was then estimated by mapping the experimentally measured FWHM onto the previously established FWHM–axial distance calibration curve.

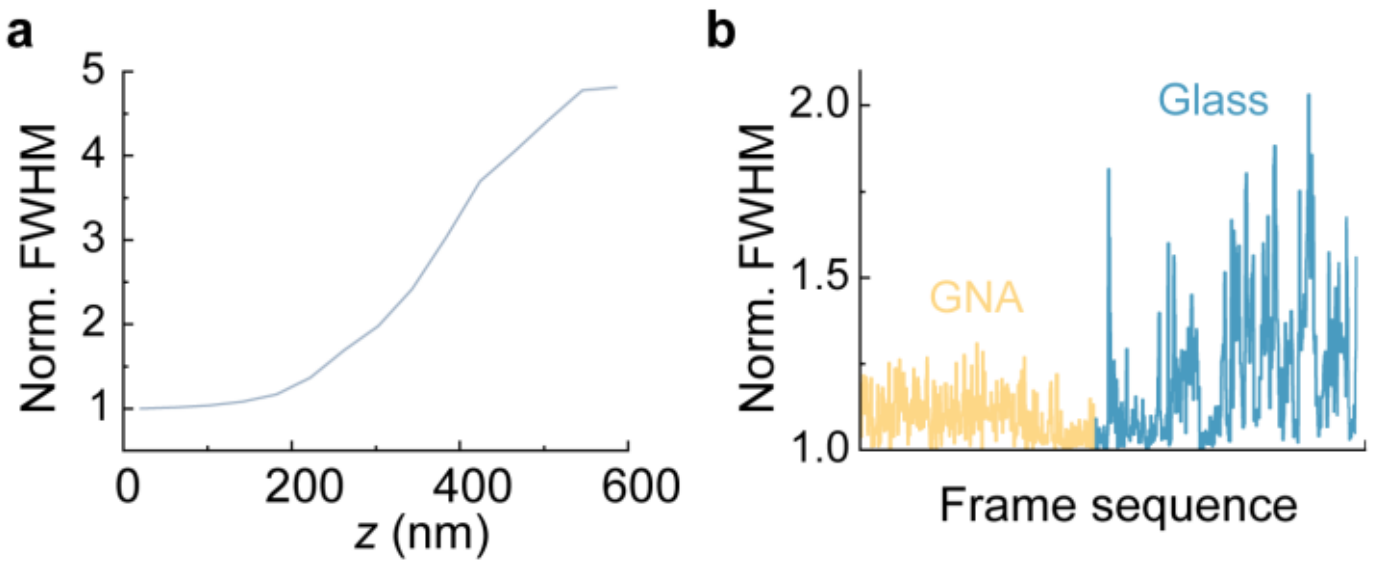

Figure S6. (a) Calibration curve for the axial (*z*-axis) localization of the UCNP within optical trap. **(b)** A representative temporal evolution of the normalized FWHM of the luminescence spot size of a single optically trapped UCNP, extracted from sequentially recorded image frames.

The uncertainty in the axial coordinate *z* is obtained by propagating the uncertainty in the measured FWHM through the simulated calibration curve. Each luminescence spot is fitted with a 2D Gaussian function to extract its FWHM and the associated fitting uncertainty ($\delta(\mathrm{FWHM})$). The corresponding uncertainty in axial position is then estimated as:

$$\delta z \approx \left| \frac{dz}{d(\mathrm{FWHM})} \right| \delta(\mathrm{FWHM}) \qquad \textbf{[Eq. S2]}$$

where $dz/d(\mathrm{FWHM})$ is obtained from the simulated calibration curve. This provides a frame-by-frame estimate of the axial localization uncertainty. The fitting of experimental data (fluorescence images at different times) leads to an average uncertainty of ${}^{\delta(\mathrm{FWHM})}/_{\mathrm{FWHM}} \approx 0.004$ i.e. uncertainty in the determination of the FWHM of 0.4%. **Figure S7** provides the calculated uncertainty in the axial coordinate as obtained from **[Eq. S2]**. We found that for an axial position of 50 nm the uncertainty we have in the axial position is close to 10 nm. Based on this analysis, the axial positions should be interpreted as simulation-assisted estimates with an uncertainty on the order of tens of nanometers, rather than absolute high-precision measurements. Importantly, the same localization procedure is

consistently applied to both trapping on glass and on the nanoantenna, so the relative comparison between both conditions remains robust.

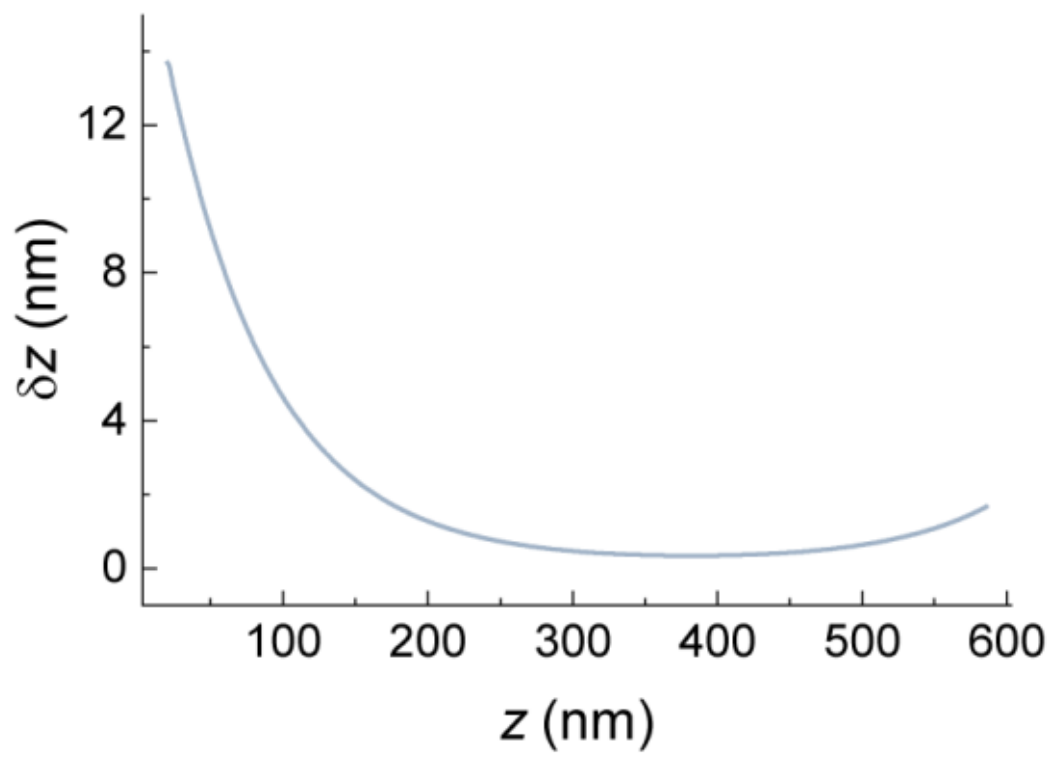

Figure S7. Estimated uncertainty in the axial position, δz, as a function of the nanoparticle axial coordinate, z. The uncertainty was calculated by propagating the frame-by-frame uncertainty in the experimentally determined fluorescence-spot FWHM through the simulated FWHM–z calibration curve, according to $\delta z \approx | dz/d(\mathrm{FWHM}) |\, \delta(\mathrm{FWHM})$. At $z \approx 50$ nm, the estimated axial-position uncertainty is approximately 10 nm.

Section S9. Simulations of the electric field enhancement

The observed enhancement in the upconversion luminescence intensity can be caused by the local enhancement in the 980 nm excitation intensity and/or by the increment in the density of states at the emitting wavelengths (Purcell effect). To determine the dominant mechanism, we have calculated the excitation (980 nm) and emission (550 nm) field distributions within the array of gold nanoantenna. Specifically, we calculated the spatial distribution of the normalized electric field intensity ($|E|^2/|E_0|^2$) under plane-wave illumination at both wavelengths (**Figure S8a, b**). At 980 nm, field enhancement reaches values of ~200 near the nanoantenna tips and extends over a volume that overlaps with the UCNP position. In contrast, at 550 nm, the enhancement is spatially localized predominantly at the nanoantenna bottom edges, with minimal overlap with the UCNP location. This spatial and spectral mismatch indicates that the emitter experiences strong enhancement at the excitation wavelength but limited modification of the local density of optical states (LDOS) at the emission wavelength, and therefore a weak Purcell effect. To further support this interpretation, we calculated the Purcell factor for an emitter positioned at z = 10 nm as a function of the distance to the antenna tip (dashed line in **Figure S8c**). The Purcell factor remains close to unity for distances larger than ~20 nm, indicating negligible radiative-rate modification in the region where the UCNP is located. In contrast, the excitation enhancement at 980 nm remains significant, reaching ~5.6× at 20 nm distance. Taken together, these results show that, within the relevant spatial region, excitation enhancement clearly dominates over radiative rate (Purcell) effects, supporting the interpretation that excitation amplification is the primary mechanism driving the observed UCNP luminescence increase.

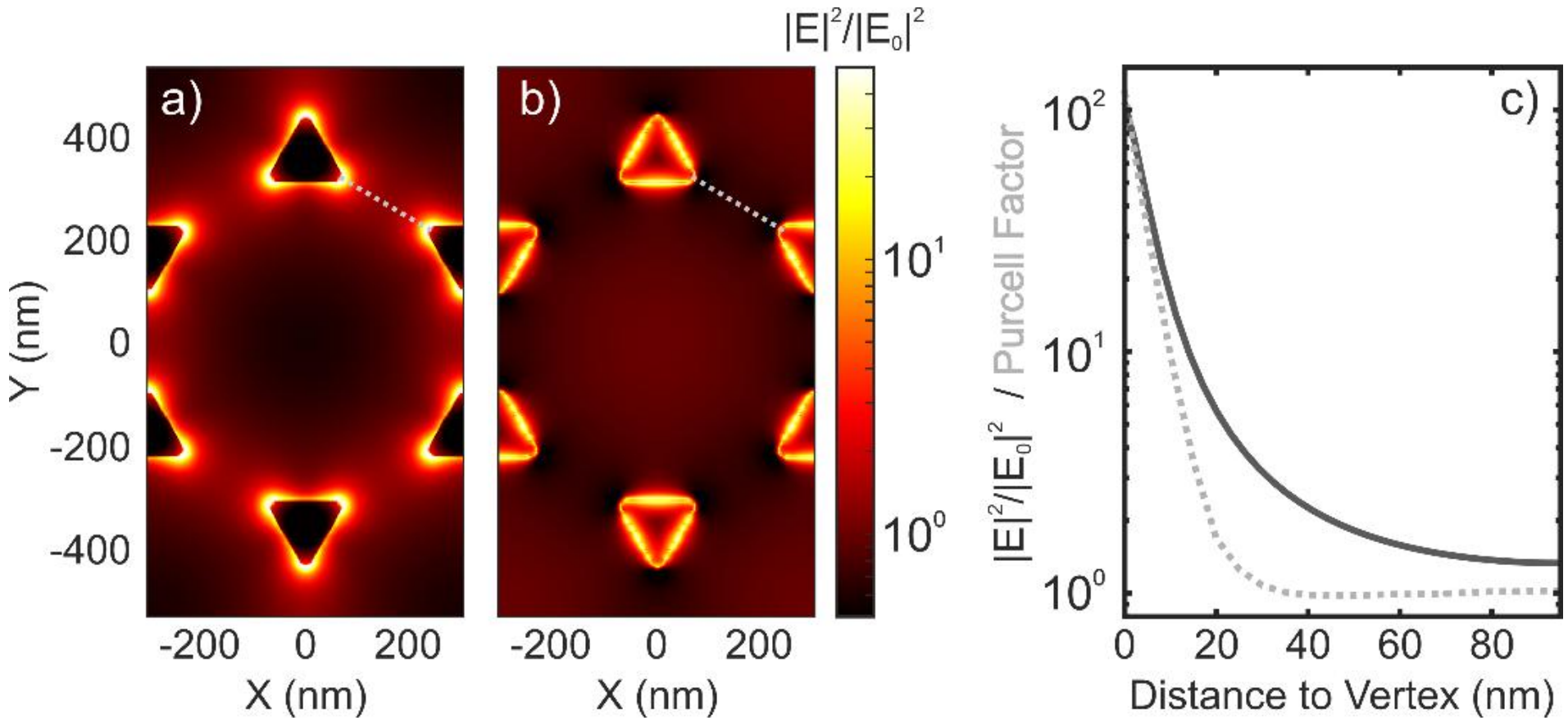

Figure S8. (a)-(b) FDTD calculations for the electric field intensity in the nanoantenna pattern under 980 nm (a) and 550 nm (b) plane wave illumination. **(c)** Comparison between electric field intensity enhancement at the excitation wavelength (black line) and Purcell factor (gray dashed line) as a function of nanoantenna tip distance.

To investigate the effect that the exact location of the 980 nm beam on the GNA array we have also performed gaussian beam simulations using a 5 µm × 5 µm area without periodic boundary conditions. Based on these simulations, it can be observed that the electric field maximum consistently occurs at the vertices of the nanoantennas, regardless of the focus position (**Figure S9a**). This indicates that the UCNP is trapped in the gap between two antennas, specifically localized at the vertex of one antenna. Nevertheless, higher excitation enhancement is found when the beam is focused between to nanoantenna, which is expected from the field enhancement being located at the vertices. The electric field intensity gradient was calculated for the case of beam focusing on the gap between two antennas and compared to the bare glass substrate (**Figure S9b**). The values for the gold nanoantennas are orders of magnitude higher than those for the glass substrate, providing robust evidence for the observed UCNP trapping.

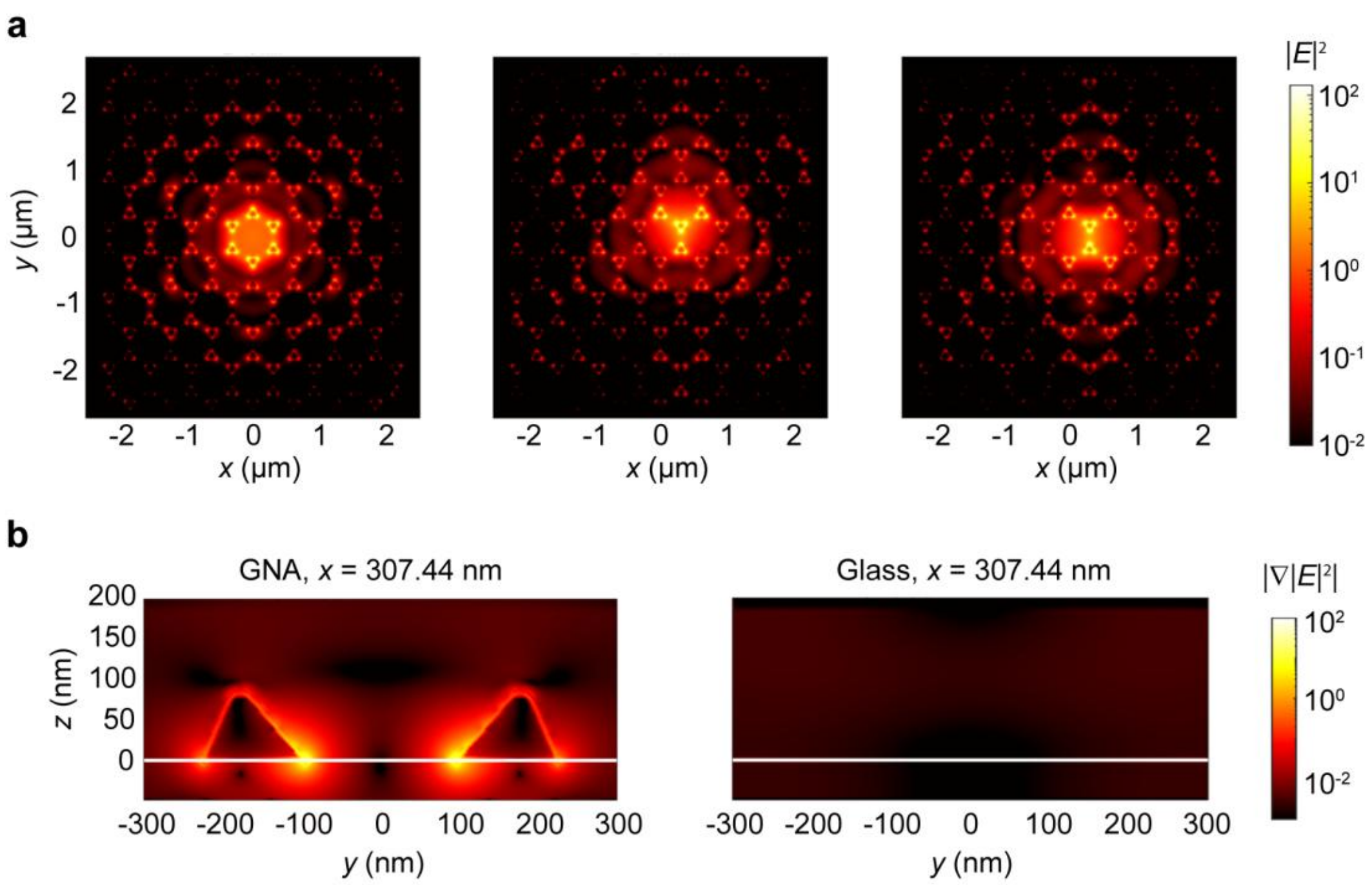

Figure S9. (a) Simulated electric field distributions for a Gaussian beam focused on the center of six nanoantennas (left), on top of a nanoantenna (middle), and on the gap between two adjacent nanoantennas (right), respectively. **(b)** Simulated electric field gradient distributions for GNA (left) and bare glass (right), respectively.

Considering the integration times used for spectral acquisition and the exposure times used for luminescence imaging, the measured enhancement should be interpreted as an averaged effect, which helps to explain the discrepancy between the simulated electric field intensity and the experimentally observed enhancement. The expected excitation intensity amplification experienced by the UCNP was characterized by a histogram constructed from both the simulated field profiles and the experimentally obtained axial positions (**Figure S10**). From this distribution, the average amplification factor was estimated to be ~4.

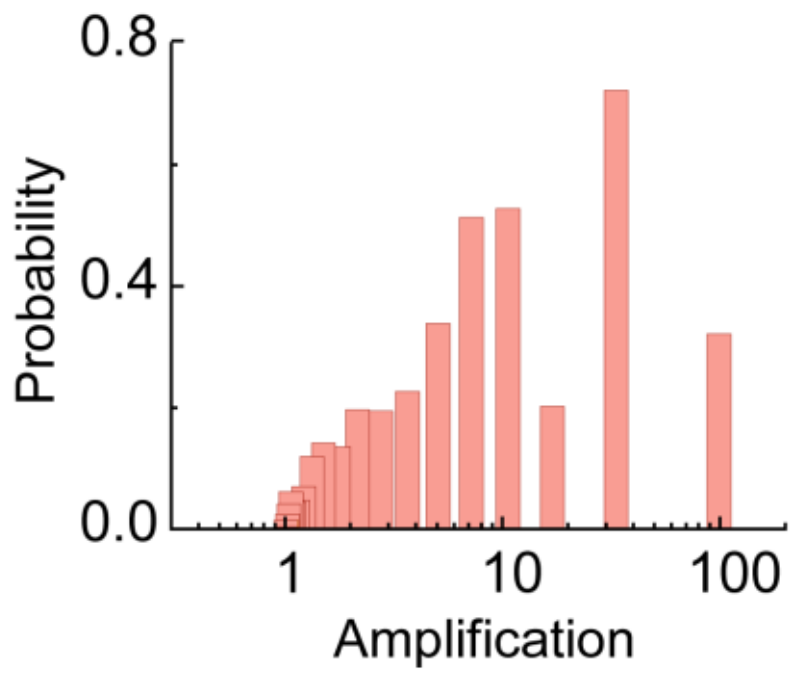

Figure S10. Histogram of the expected amplification of excitation intensity experience by the UCNP.

Section S10. Measurement of plasmonic heating

The selected literature reporting measurement of the plasmonic heating based on fluorescence thermometry and anti-Stokes thermometry are summarized in **Table S1**. Similar to this work, fluorescence thermometry primarily reflects the localized temperature of the medium in vicinity of the surface plasmonic nanostructure. Our experimentally obtained value is comparable to the reported values. In contrast, the anti-Stokes method probes the temperature of the electrons within the plasmonic structure, which is more closely related to the surface temperature of the nanostructure than that of the surrounding medium, thereby resulting in a higher apparent heating rate.

Table S1. Selected literature reporting measured near-surface temperature rise induced by plasmonic heating

Plasmonic structure	Methods	Maximum extinction (nm)	Irradiation source	Measured heating rate	Ref.
Gold nanowire (250 nm × 30 nm)	Fluorescence polarization anisotropy	-	775 nm (100×, NA 1.25)	0.15 K/mW	7
Gold nanoaperture (40 nm gap)	Fluorescence spectroscopy (intensity, diffusion time, lifetime)	-	1064 nm (40×, NA 1.3)	4 K/(mW μm^{-2})	8
Gold nanosphere (100 nm)	Fluorescent melting fingerprints	550 nm	1064 nm (100×, NA 1.4)	0.452 K/mW	9
Gold nanosphere (200 nm)	Fluorescent melting fingerprints	527 nm	1064 nm (100×, NA 1.4)	4.1 K/(mW μm^{-2})	10
Gold nanosphere (80, 150 nm)	Fluorescent melting fingerprints	550 nm (80 nm size) 614 nm (150 nm size)	1064 nm (100×, NA 1.4)	80 nm size: 0.493 K/mW 150 nm size: 0.573 K/mW	11
Gold nanorod (27.56 nm × 30 nm)	Spectral shift of CdSe quantum dots	780 nm	800 nm (beam radius: 1.12 μm)	0.8 K/mW	12
127-nm thick sliver coated polystyrene bead	Luminescence intensity of Eu^{3+} doped film	480 nm	Ar laser (476.5, 488, 514 nm)	0.11 K/mW	13
Gold nanoaperture (70, 100, 130 nm-thick film)	Luminescence intensity ratio of Er^{3+} doped single nanoparticle	-	980 nm (100×)	70-nm thick: 0.64 K/mW 100-nm thick: 0.37 K/mW 130-nm thick: 0.25 K /mW	14
Gold nanoantenna (140 nm)	Luminescence intensity ratio of Er^{3+} doped single nanoparticle	820 nm	980 nm (100×, NA 1.4)	0.6 K/mW	This work
Gold nanodisk (174 nm)	Anti-Stokes Raman spectroscopy	785 nm	785 nm (beam radius: 1.1 μm)	8.7 K/mW	15
Gold bowtie nanoantenna (varying length: 120, 150, 180, with fixed width of 70 nm and height of 30 nm)	Anti-Stokes Raman spectroscopy	880 nm (120 nm length) 1050 nm (150 nm length) 1150 nm (180 nm length)	1064 nm	120 nm length: 1.6 K/mW 150 nm length: K/mW 180 nm length: 11 K/mW	16
Gold nanorod (25 nm × 50 nm)	Anti-Stokes Raman spectroscopy	625 nm	633 nm	667.1 K /mW	17
Gold nanospheres (80 nm)	Anti-Stokes Raman spectroscopy	560 nm	532 nm (60×, NA 1)	on glass: 62 K/(mW μm^{-2})	18

Plasmonic structure	Methods	Maximum extinction (nm)	Irradiation source	Measured heating rate	Ref.
				on sapphire: 51 K/(mW μm^{-2})<br>on graphene: 77 K/(mW μm^{-2})	
Gold nanodisk (65, 100, 157 nm in diameter)	Anti-Stokes Raman spectroscopy	585 nm (65 nm)<br>610 nm (100 nm)<br>725 nm (157 nm)	647 nm (NA 1.1)	65 nm: 292 K/(mW μm^{-2})<br>100 nm: 769 K/(mW μm^{-2})<br>157 nm: 426 K/(mW μm^{-2})	19
Gold nanodisk array	Anti-Stokes Raman spectroscopy	600 nm	533 nm (20×, NA 0.4)	~ 33 K/mW	20

Section S11. Simulations of the temperature map

The temperature maps obtained in this work were simulated by solving numerically the heat transfer **Equation S1**. This equation relates the space and time partial derivatives of the local temperature in an object with the energy deposited by heat sources and energy loss into the surrounding medium.[21] The heat transfer equation for inhomogeneous media reads as:

$$\rho(\vec{r})c(\vec{r})\frac{\partial T(\vec{r},t)}{\partial t} = \nabla[\kappa(\vec{r})\nabla T(\vec{r},t)] + q_v(\vec{r},t) \qquad [\mathbf{Eq.\,S4}]$$

Here, T is the local temperature (K), t the time (s), ρ is the mass density (kg·m^{-3}), c is the specific heat capacity (J/kg·K), κ is the thermal conductivity (W·m^{-1}·K^{-1}), and q_v is the externally supplied power per unit volume (W·m^{-3}). **Equation S1**, besides the appropriate boundary conditions, is solved numerically by a 3D-finite difference scheme using the Douglas-Gunn method. This scheme provides a stable algorithm that is second order accurate in both time and space derivatives.[22, 23]

The time evolution of the temperature map of the region of interest is computed by a time-marching scheme, where the temperature at each spatial point and at a given time is calculated from the 3D spatial distribution of the temperature at the previous time step. Denoting the magnitudes at the time steps n and $n + 1$ by superscripts, respectively, and Δt represents the time step, **Eq. S4** can be written as:

$$\left[1 - \frac{\Delta t}{2\rho c}\delta_{xx} - \frac{\Delta t}{2\rho c}\delta_{yy} - \frac{\Delta t}{2\rho c}\delta_{zz}\right]T^{n+1} =$$
$$\left[1 + \frac{\Delta t}{2\rho c}\delta_{xx} + \frac{\Delta t}{2\rho c}\delta_{yy} + \frac{\Delta t}{2\rho c}\delta_{zz}\right]T^{n} + \frac{\Delta t}{2}(F^n + F^{n+1}) \qquad [\mathbf{Eq.\,S5}]$$

This equation is obtained by implementing a Crank-Nicolson scheme to increase accuracy and ensure stability. The spatial differential operators are defined as follows:

$$\delta_{xx}T \equiv \frac{\partial}{\partial x}\left[\kappa\frac{\partial T}{\partial x}\right],\ \delta_{yy}T \equiv \frac{\partial}{\partial y}\left[\kappa\frac{\partial T}{\partial y}\right],\ \delta_{zz}T \equiv \frac{\partial}{\partial z}\left[\kappa\frac{\partial T}{\partial z}\right]$$

and the magnitude is defined as $F \equiv \frac{q_v}{\rho c}$.

Eq. S5 is then converted to a 3D-finite difference equation, where it can be solved by the Douglas-Gunn method using alternative direction implicit (ADI) techniques, resulting in three tridiagonal systems, which can be numerically solved in a very efficient way using the Thomas algorithm.[24] Also, for solving the differential **Eq. S4**, it is necessary to apply appropriate boundary conditions.

The splitting of the **Eq. S5** following the Douglas-Gunn method for 3D geometries results in:

$$\left(1 - \frac{r}{2}\delta_{xx}\right)T_{ijk}^{n+1/3} = \left(1 + \frac{r}{2}\delta_{xx} + r\delta_{yy} + r\delta_{zz}\right)T_{ijk}^{n} + \frac{\Delta t}{2}(F^n + F^{n+1}) \qquad [\mathbf{Eq.\,S6a}]$$

$$\left(1 - \frac{r}{2}\delta_{yy}\right)T_{ijk}^{n+2/3} = T_{ijk}^{*} - \frac{r}{2}\delta_{yy}T_{ijk}^{n} \qquad [\mathbf{Eq.\,S6b}]$$

$$\left(1 - \frac{r}{2}\delta_{zz}\right)T_{ijk}^{n+1} = T_{ijk}^{n+2/3} - \frac{r}{2}\delta_{zz}T_{ijk}^{n} \qquad [\mathbf{Eq.\,S6c}]$$

where T_{ijk}^{n} denotes the temperature at the nodal point ($i\,j,k$) at time step n, T_{ijk}^{n+1} is the temperature at time ($t + \Delta t$), and $T_{ijk}^{n+1/3}$ and $T_{ijk}^{n+2/3}$ are the temperature at fictitious time steps $n + 1/3$ and $n + 2/3$, respectively. For the sake of clarity, we have defined the parameter r as: $r \equiv \frac{\Delta t}{\rho c}$. Thus, **Eqs. S6a-S6c** provide a marching scheme for calculating the temperature map at time ($t + \Delta t$), from a given temperature distribution at the previous time t, being the algorithm efficient and stable.

To check the accuracy of the simulations of the heating provided by the nanoobjects, we compared the results with known closed form formulae.[25] For this purpose, we simulated the temperature maps of a single nanoparticle heater, and we have found agreement with the formulae reported by Govorov et al..[26] Also, the algorithm here described was checked in the case of more than one nanoheater, and the numerical simulations were in accordance with data provided by nanoparticle arrays.[27]

Our thermal simulations do consider the gaussian profile of the excitation beam together with the simultaneous heating of multiple nanoantennas within the illuminated area. The number of GNA effectively illuminated by the gaussian beam as well as the actual fraction of energy deposited into each GNA is expected to be strongly dependent on the exact location of the 980 nm beam within the GNA array. Two excitation scenarios are considered, as summarized in **Figure S11**: Scenario A, where the beam is focused directly on top of a nanoantenna, and Scenario B, where the Gaussian beam is focused at the center of the hexagonal array. In both cases, we assumed a beam waist of 500 nm, consistent with the experimental trapping conditions.

Thermal simulations clearly demonstrate the importance of collective heating. When the beam is focused on a nanoantenna (Scenario A), the central nanoantenna absorbs ~40% of the total absorbed power, while the three nearest neighbors each absorb ~15% (**Figure S11a**). The resulting thermal map (**Figure S11b**) shows a dominant hot spot at the central antenna together with three additional hot regions corresponding to the nearest neighbors. The temperature between these hot spots does not decrease down to 20 ºC (substrate temperature) directly evidencing collective heating within the array. The calculated axial thermal profiles for different absorbed powers are shown in **Figure S11c**. From these simulations, we find that the experimentally measured temperature increase (~40 °C) is reproduced for an absorbed power of 0.027 mW at the central nanoantenna that corresponds to a total absorbed power (accounting for the absorption of all the nanoantenna) of 0.067 mW that is 0.1 % of the 980 nm laser power (60 mW). For such an absorbed pump power the numerical simulations (**Figure S11b**) estimate ∼60 ºC for the temperature increment at the gold-water interface.

A similar analysis can be performed for Scenario B in which the 980 nm laser beam is focused on the center of the hexagonal. In this case, the six surrounding nanoantennas absorb the same fractions of the incident power (~14% each), while more distant nanoantennas absorb negligibly (**Figure S11d**). In this case, the calculated thermal map exhibits six equivalent hot spots and a much stronger collective thermal accumulation effect (**Figure S11e**). Indeed, the whole hexagonal array is heated. Matching the experimentally measured temperature increase (**Figure S11f)** now requires a lower absorbed power per nanoantenna (~0.018 mW). In this case, the total absorbed laser power is calculated to be 1.28 mW that corresponds to a fraction of 0.2% of the total 980 nm power (60 mW). In this case the thermal map (**Figure S11e**) reveals that the temperature increment induced at the gold-water interface is ≈ 45 ºC.

In our experiments, we believe the system operates closer to Scenario A because the laser position was experimentally optimized to maximize both trapping stability and UCNP luminescence enhancement, conditions that occur when the excitation field is most efficiently coupled to a nanoantenna hotspot.

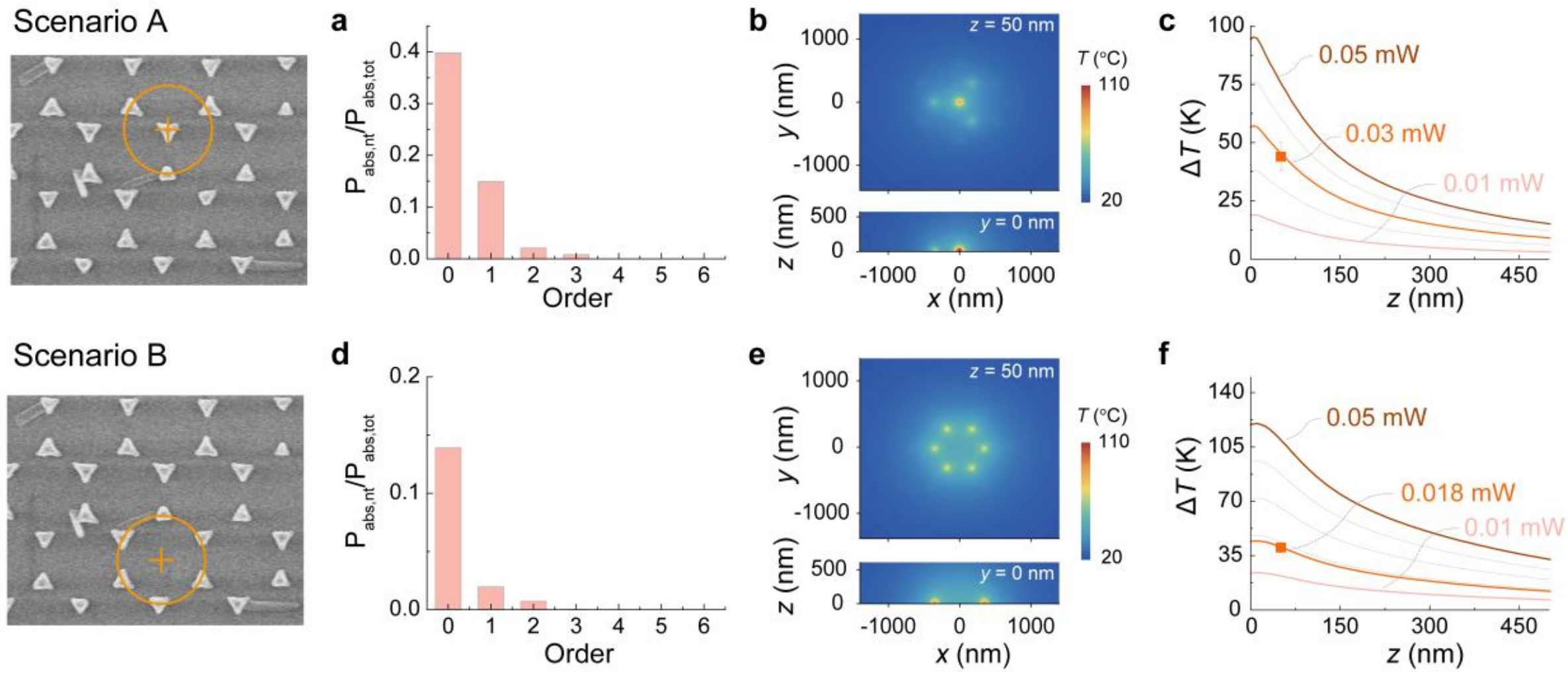

Figure S11. Collective heating effects in the gold nanoantenna array under 980 nm excitation. **(a–c)** Scenario A: the excitation beam is centered on a single nanoantenna. **(a)** Relative absorbed power as a function of nanoantenna order. **(b)** Simulated temperature maps (top and cross-sectional views) showing thermal accumulation from neighboring nanoantennas. **(c)** Axial temperature profiles calculated for different absorbed powers. **(d–f)** Scenario B: the excitation beam is centered at the middle of the hexagonal array. **(d)** Relative absorbed power distribution among illuminated nanoantennas. **(e)** Simulated temperature maps evidencing stronger collective heating. **(f)** Corresponding axial temperature profiles for different absorbed powers. Black squares indicate the experimentally measured temperature increase of the UCNP (~40 °C at 50 nm above the substrate), used to estimate the absorbed power and the corresponding nanoantenna temperature.

Section S12. Thermal stability of gold nanoantenna

Previous works showed that the morphology of the gold nanoantennas used in this work can be modified by high-temperature annealing.[1] However, those changes were observed at temperatures around 450 °C, which are substantially higher than the local temperatures reached in our experiments (estimated to be approximately of the order of 100ºC as it is described in main text). We therefore do not expect significant thermally induced reshaping of the plasmonic nanostructures under our experimental conditions. To explore the possible presence of re-shaping effects, we performed additional experiments to systematically examine the morphology of the nanoantennas after thermal annealing at different temperatures. As shown in **Figure S12a**, the overall nanoantenna geometry remains well preserved after annealing at temperatures up to 300 °C. A more detailed analysis of the SEM images, including the size histograms shown in **Figure S12b**, reveals only a slight reduction in side length with increasing annealing temperature. At 100 °C, the estimated reduction is approximately 6% (**Figure S12c**).

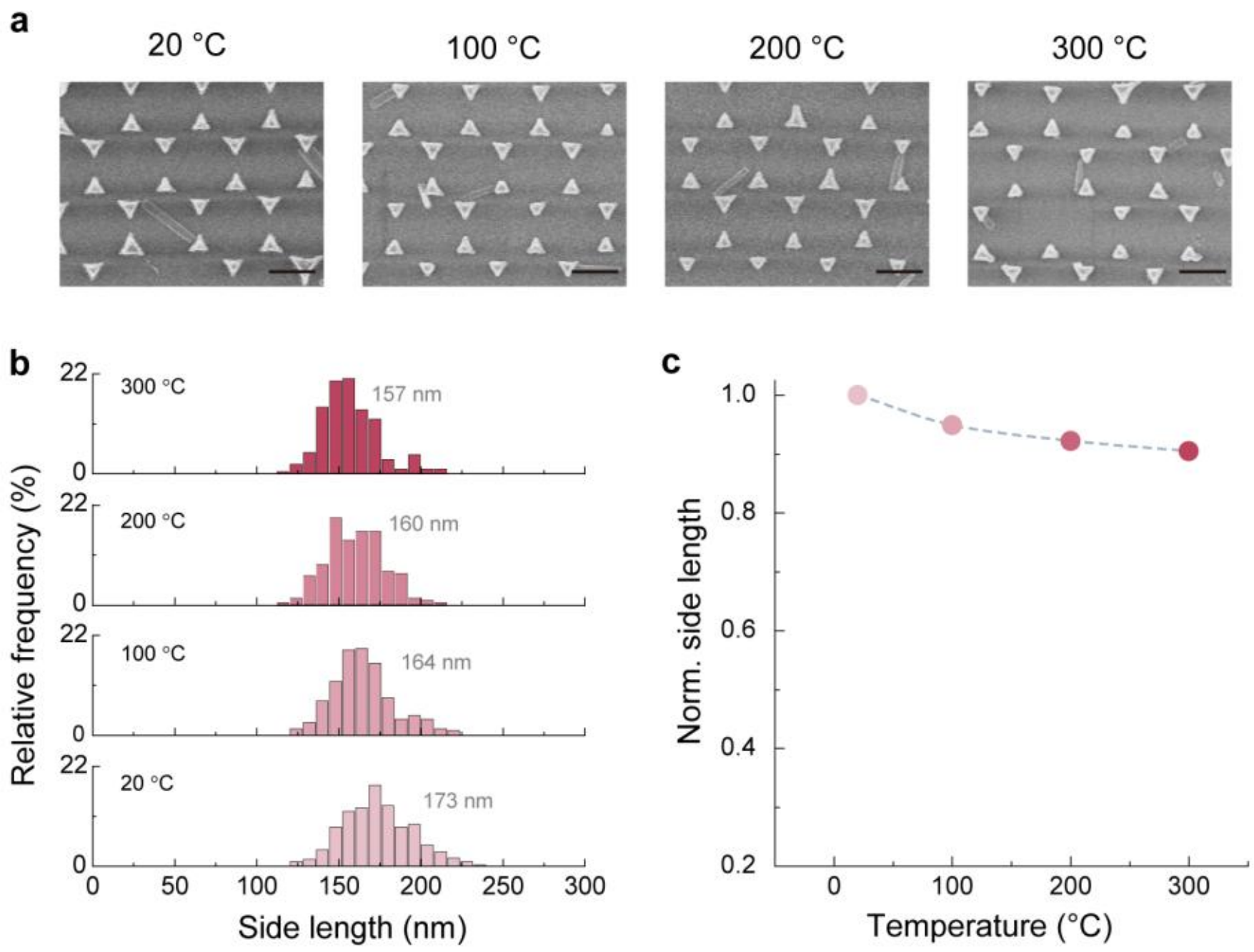

Figure S12. (a) Representative SEM images of the nanoantenna array before annealing (20 °C) and after thermal treatment at 100, 200, and 300 °C. The overall morphology and spatial arrangement of the nanoantennas remain largely preserved over the investigated temperature range. Scale bars: 500 nm. **(b)** Histograms of the nanoantenna side length extracted from the SEM images, showing only minor changes in the size distribution after annealing. The mean side lengths are indicated in each histogram. **(c)** Normalized nanoantenna side length as a function of annealing temperature. Dots are experimental data and dashed line is a guide for the eyes. A slight decrease in side length is observed with increasing temperature, reaching a reduction of approximately 6% at 100 °C and less than 10% even after annealing at 300 °C. These results confirm the high thermal stability of the nanoantenna geometry under temperatures well above those expected during the optical trapping experiments.

We further evaluated the impact of this size reduction on the optical response of the nanoantenna by means of numerical simulations. As shown in **Figure S13**, a 10% decrease in nanoantenna size (larger than the expected reduction caused by a 100 ºC annealing) produces no significant change in either the position or the magnitude of the field enhancement at 980 nm. These results indicate that although thermal annealing may induce minor morphological changes, their effect on the plasmonic performance of the nanoantenna is negligible under the conditions relevant to our experiments. This conclusion is also supported by the experimental reproducibility of the trapping measurements. We consistently observe similar trapping behavior when repeatedly positioning UCNPs on both glass substrates and

plasmonic arrays. If the nanoantennas were significantly damaged or reshaped during the experiments, a much larger variability in trapping performance would be expected. Since this is not observed, the reproducibility of the measurements provides strong indirect evidence that the gold nanoantennas remain structurally stable under our working conditions.

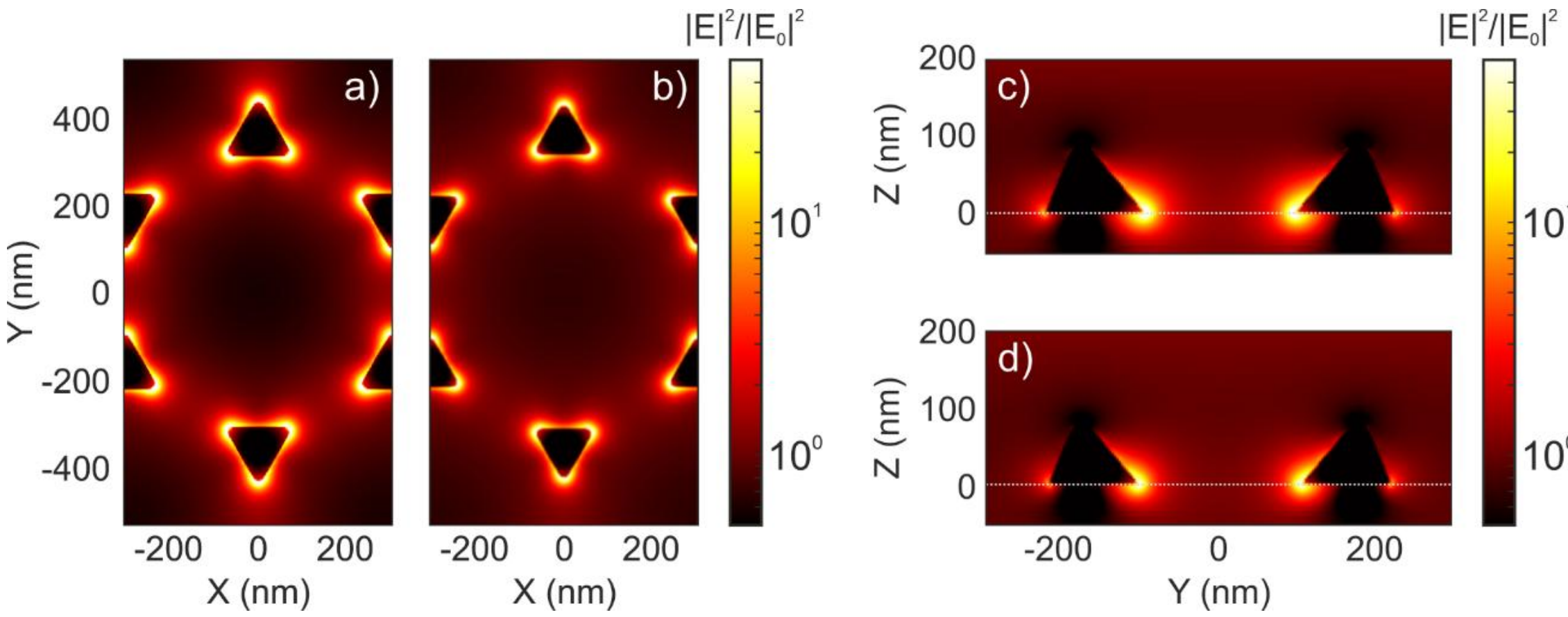

Figure S13. Simulated near-field intensity distribution of the 980 nm excitation for a pyramid-like nanoantenna array. **(a)** and **(c)** correspond to the top and cross-sectional views of a pre-annealed array, respectively, whereas **(b)** and **(d)** correspond to the top and cross-sectional views of the array with a 10% size reduction, respectively. No significant changes in the near-field distribution are observed.

Section S13. Thermal Gradient and effective thermophoretic force

The calculated thermal map allows for an estimation of the axial thermal profiles, the thermal gradients, and the effective thermophoretic force. **Figure S14a** includes the axial dependence of the temperature of the GNA (at its apex) for the situation in which the 980 nm laser beam is focused on top of the GNA and the absorbed pump power by the GNA is 0.027 mW. **Figure S14b** shows the axial dependence of the thermal gradient getting a value of dT/dz = -0.23 K/nm at z = 50 nm (where the UCNP is trapped). **Figure S14c** shows the effective thermophoretic force that can be calculated applying expression $F_{Th} = -k_B \cdot T \cdot S \cdot \nabla T$ assuming (as a conservative upper-bound value for dielectric nanoparticles) a value of $S = 0.1\ \mathrm{K}^{-1}$. In these conditions the effective thermophoretic force estimated at z = 50 nm is 0.1 pN.

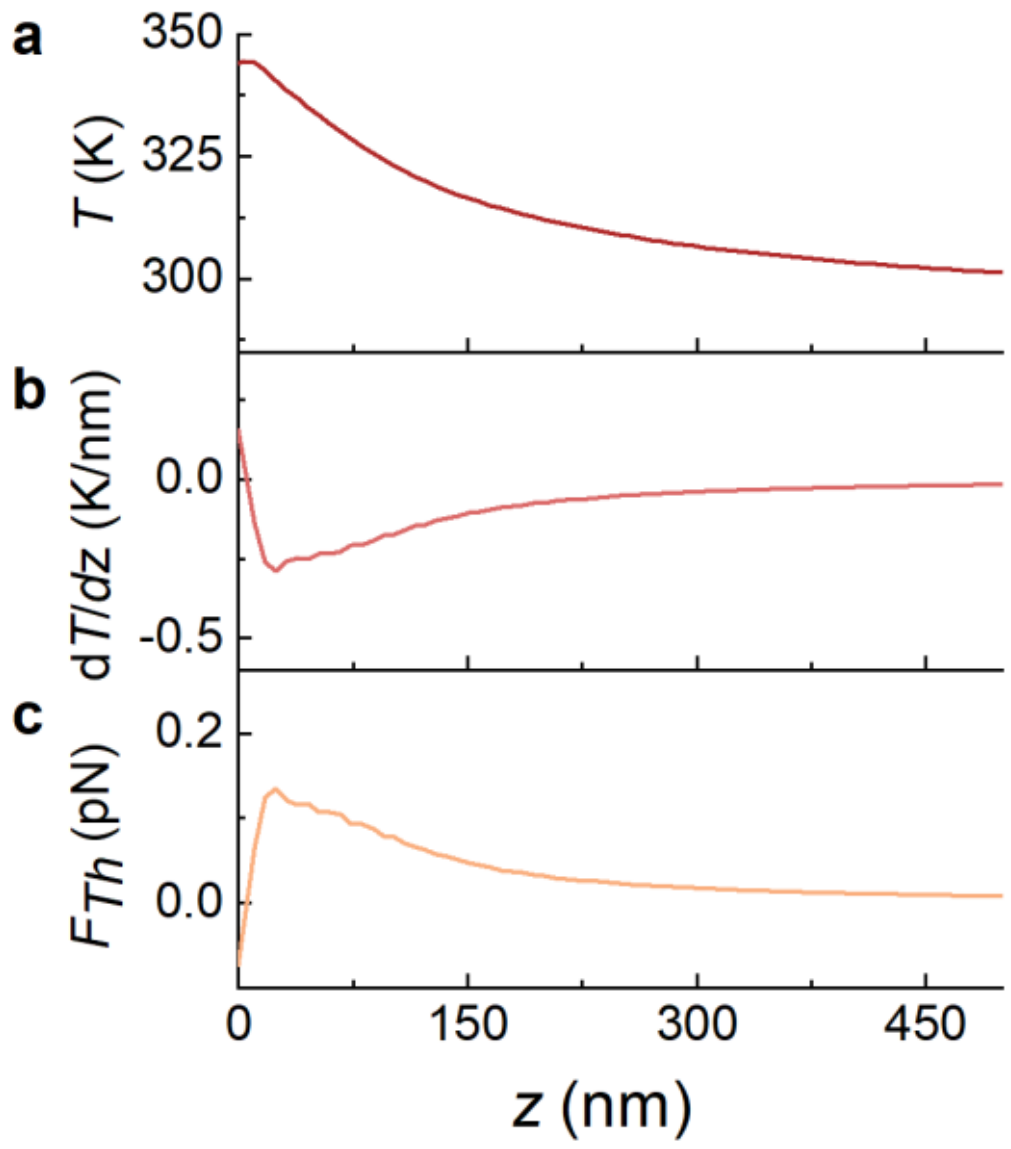

Figure S14. Estimation of the thermophoretic force acting on the trapped UCNP. **(a)** Calculated axial temperature profile above the apex of the central GNA under focused 980 nm excitation. **(b)** Corresponding axial temperature gradient (dT/dz) generated around the plasmonic nanoantenna as obtained by differentiating the thermal profile included in (a). At the experimentally observed trapping position of the UCNP ($z \approx 50$ nm), the local thermal gradient is approximately −0.23 K/nm. **(c)** Effective thermophoretic force calculated from the simulated temperature and thermal-gradient profiles using a conservative upper-bound value of the Soret coefficient for dielectric nanoparticles.

Section S14. Optical, electrostatic and effective thermophoretic potentials

In this section we analyze the balance between optical trapping, thermophoresis, and electrostatic repulsion to clarify the origin of the equilibrium trapping position and to determine whether the UCNP is expected to contact the gold nanoantenna. In our experiments, the UCNP is trapped close to a gold nanoantenna deposited on a glass substrate and immersed in distilled water. Under these conditions, we consider that the particle experiences three main contributions: the optical trapping force, the thermophoretic force, and the electrostatic interaction with the substrate. Consequently, the total effective potential experienced by the UCNP can be written as:

$$U_{eff} = U_s + U_{Th} + U_{opt} \qquad \textbf{[Eq. S7]}$$

where U_s, U_{Th} and U_{opt} are the potential associated to the substrate-UCNP electrostatic interaction, to the electrophoresis force and to the optical force. The optical potential is attractive and strongly localized near the nanoantenna due to the plasmon-enhanced electromagnetic field at 980 nm. In contrast, both the thermophoretic and electrostatic contributions are repulsive and extend over distances of several tens to hundreds of nanometers away from the substrate.

Regarding the electrostatic contribution, the gold nanoantennas are deposited on a non-treated glass substrate that forms the bottom of the trapping chamber. Under aqueous conditions, silanol groups (SiOH) at the glass surface partially deprotonate, leading to negatively charged siloxide groups (SiO^-) at the interface.[28] Since the UCNPs used in this work are also negatively charged (ζ=-37 mV), a repulsive electrostatic interaction appears between the particle and the substrate. This interaction can be described by:

$$U_s(z) = U_{s,0} \cdot e^{-z/\lambda_D} \qquad \textbf{[Eq. S8]}$$

where $U_{s,0}$ is the repulsive energy at contact and λ_D is the Debye length. Under our experimental conditions (distilled water in equilibrium with atmospheric CO_2), previous studies indicate $\lambda_D \approx 100\ nm$). Importantly, this electrostatic repulsion is also consistent with the experimental observation that, on bare glass, the trapped UCNP remains centered approximately 100 nm above the substrate (Figure 3g). The shape of $U_s(z)$ potential curve is shown in **Figure S13**.

As it is stated in the main text, the "effective" thermophoretic force can be written as:

$$F_{\mathrm{Th}}(z) = -k_B T(z)\, S\, \nabla T(z) \qquad \textbf{[Eq. S9]}$$

where k_B is the Boltzmann constant, $T(z)$ is the local temperature, S is the Soret coefficient, and $\nabla T(z)$ is the thermal gradient. The axial component of the thermal gradient was obtained from the thermal simulations (Figure 5 in main text). Integrating this force along the axial coordinate yields

$$U_{TH}(z) = -\int F_{Th}(z)dz = \int_{\infty}^{z} k_B \cdot T(z) \cdot S \cdot \nabla T(z) \cdot dz = \int_{\infty}^{z} k_B \cdot T(z) \cdot S \cdot \frac{dT(z)}{dz} \cdot dz = \frac{1}{2} k_B \cdot S \cdot [T(z)^2 - T_{amb}^2] \qquad \textbf{[Eq. S10]}$$

where T_{amb} = 293 K is the chamber temperature. The $U_{TH}(z)$ normalized curve is also shown in **Figure S15**. The resulting thermophoretic potential shows a spatial dependence similar to that of electrostatic repulsion, extending over distances much larger than the optical near field. Interestingly, very close to the nanoantenna surface the thermophoretic force becomes slightly negative due to heat dissipation through the glass substrate, which acts as a thermal sink. However, for distances larger than approximately 10 nm, the thermophoretic force becomes repulsive, pushing the UCNP away from the hottest region of the nanoantenna.

The optical force acting on a nanoparticle much smaller than the excitation wavelength is proportional to the intensity gradient:

$$F_{opt,z}(z) = \frac{1}{2}\alpha_{NP}\frac{\partial}{\partial z}|E(z)|^2 \qquad \textbf{[Eq. S11]}$$

where α_{NP} is the polarizability of the UCNP and E is the local electric field. The electric field distribution around the nanoantenna was obtained from the electromagnetic simulations shown in **Figure 1d** of the main text. The corresponding optical potential was then calculated as:

$$U_{opt}(z) = -\int_{\infty}^{z} F_{opt,z}(z)\,dz \qquad \textbf{[Eq. S12]}$$

The z dependence of the optical potential, obtained from numerical simulations of the electric field distribution around the nanoantenna, is also shown in **Figure S14**. In contrast to the electrostatic and thermophoretic potentials, the optical potential is strongly localized near the gold nanoantenna, where plasmon-induced enhancement of the 980 nm field occurs.

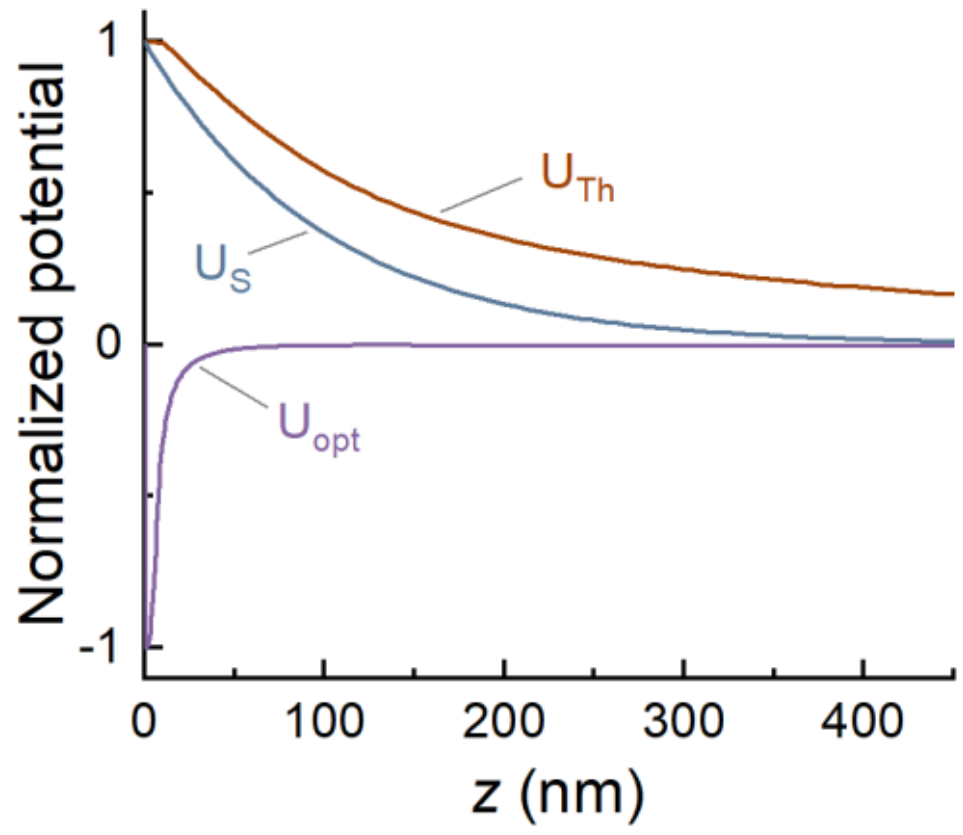

Figure S15. Axial dependence of the normalized electrostatic, thermophoretic and optical potentials.

Calculations included in **Figure S15** explain why the equilibrium trapping position does not occur at the gold surface itself. Instead, the observed trapping position (z = 50 nm) results from the balance between the attractive optical force and the combined repulsive electrostatic and thermophoretic interactions. Consequently, the UCNP is not expected to physically contact the gold nanoantenna. We should add at this point that the experimental evidence showing stable trapping of the UCNP on the gold nanoantenna array provided in main text indicate that the optical potential dominates over repulsive potentials (electrostatic and thermophoretic).

References

1. Viaña, J. M.; Romero, M.; Lozano, G.; Míguez, H., Nanoantennas Patterned by Colloidal Lithography for Enhanced Nanophosphor Light Emission. *ACS Applied Nano Materials* **2022,** *5* (11), 16242-16249.
2. Sun, J.; Tang, C.-j.; Zhan, P.; Han, Z.-l.; Cao, Z.-S.; Wang, Z.-L., Fabrication of Centimeter-Sized Single-Domain Two-Dimensional Colloidal Crystals in a Wedge-Shaped Cell under Capillary Forces. *Langmuir* **2010,** *26* (11), 7859-7864.
3. Mendez-Gonzalez, D.; Laurenti, M.; Latorre, A.; Somoza, A.; Vazquez, A.; Negredo, A. I.; López-Cabarcos, E.; Calderón, O. G.; Melle, S.; Rubio-Retama, J., Oligonucleotide Sensor Based on Selective Capture of Upconversion Nanoparticles Triggered by Target-Induced DNA Interstrand Ligand Reaction. *ACS Applied Materials & Interfaces* **2017,** *9* (14), 12272-12281.
4. Marciniak, L.; Waszniewska, K.; Bednarkiewicz, A.; Hreniak, D.; Strek, W., Sensitivity of a Nanocrystalline Luminescent Thermometer in High and Low Excitation Density Regimes. *The Journal of Physical Chemistry C* **2016,** *120* (16), 8877-8882.
5. Neuman, K. C.; Block, S. M., Optical trapping. *Review of scientific instruments* **2004,** *75* (9), 2787-2809.
6. Wolf, E., Electromagnetic Diffraction in Optical Systems. I. An Integral Representation of the Image Field. *Proceedings of the Royal Society of London. Series A, Mathematical and Physical Sciences* **1959,** *253* (1274), 349-357.
7. Baffou, G.; Kreuzer, M. P.; Kulzer, F.; Quidant, R., Temperature mapping near plasmonic nanostructures using fluorescence polarization anisotropy. *Opt. Express* **2009,** *17* (5), 3291-3298.
8. Jiang, Q.; Rogez, B.; Claude, J.-B.; Baffou, G.; Wenger, J., Temperature Measurement in Plasmonic Nanoapertures Used for Optical Trapping. *ACS Photonics* **2019,** *6* (7), 1763-1773.
9. Bendix, P. M.; Reihani, S. N. S.; Oddershede, L. B., Direct Measurements of Heating by Electromagnetically Trapped Gold Nanoparticles on Supported Lipid Bilayers. *ACS Nano* **2010,** *4* (4), 2256-2262.
10. Ma, H.; Bendix, P. M.; Oddershede, L. B., Large-Scale Orientation Dependent Heating from a Single Irradiated Gold Nanorod. *Nano Letters* **2012,** *12* (8), 3954-3960.
11. Jørgensen, J. T.; Norregaard, K.; Tian, P.; Bendix, P. M.; Kjaer, A.; Oddershede, L. B., Single Particle and PET-based Platform for Identifying Optimal Plasmonic Nano-Heaters for Photothermal Cancer Therapy. *Scientific Reports* **2016,** *6* (1), 30076.
12. Li, C.; Gan, X.; Li, X.; Gu, M., Dynamic microscale temperature gradient in a gold nanorod solution measured by diffraction-limited nanothermometry. *Applied Physics Letters* **2015,** *107* (12).
13. Cesca, T.; Perotto, G.; Pellegrini, G.; Michieli, N.; Kalinic, B.; Mattei, G., Rare-earth fluorescence thermometry of laser-induced plasmon heating in silver nanoparticles arrays. *Scientific Reports* **2018,** *8* (1), 13811.
14. Toodeshki, E. H.; Frencken, A. L.; van Veggel, F. C. J. M.; Gordon, R., Thermometric Analysis of Nanoaperture-Trapped Erbium-Containing Nanocrystals. *ACS Photonics* **2024,** *11* (4), 1390-1395.
15. Xie, X.; Cahill, D. G., Thermometry of plasmonic nanostructures by anti-Stokes electronic Raman scattering. *Applied Physics Letters* **2016,** *109* (18).
16. Jones, S.; Andrén, D.; Karpinski, P.; Käll, M., Photothermal Heating of Plasmonic Nanoantennas: Influence on Trapped Particle Dynamics and Colloid Distribution. *ACS Photonics* **2018,** *5* (7), 2878-2887.
17. Carattino, A.; Caldarola, M.; Orrit, M., Gold Nanoparticles as Absolute Nanothermometers. *Nano Letters* **2018,** *18* (2), 874-880.
18. Barella, M.; Violi, I. L.; Gargiulo, J.; Martinez, L. P.; Goschin, F.; Guglielmotti, V.; Pallarola, D.; Schlücker, S.; Pilo-Pais, M.; Acuna, G. P.; Maier, S. A.; Cortés, E.; Stefani, F. D., In Situ Photothermal Response of Single Gold Nanoparticles through Hyperspectral Imaging Anti-Stokes Thermometry. *ACS Nano* **2021,** *15* (2), 2458-2467.
19. Ezendam, S.; Nan, L.; Violi, I. L.; Maier, S. A.; Cortés, E.; Baffou, G.; Gargiulo, J., Anti Stokes Thermometry of Plasmonic Nanoparticle Arrays. *Advanced Optical Materials* **2024,** *12* (3), 2301496.

20. Li, P.; Askes, S. H. C.; del Pino Rosendo, E.; Ariese, F.; Ramanan, C.; von Hauff, E.; Baldi, A., Nanoscale Thermometry of Plasmonic Structures via Raman Shifts in Copper Phthalocyanine. *The Journal of Physical Chemistry C* **2023,** *127* (20), 9690-9698.
21. Bergman, T. L., *Fundamentals of heat and mass transfer*. John Wiley & Sons: 2011.
22. Douglas Jr, J.; Gunn, J. E., A general formulation of alternating direction methods. *Numèrische mathèmatik* **1964,** *6* (1), 428-453.
23. Duglus, I., On the numerical integration of Uxx+ Uyy= Ut by implicit methods. *J. Soc. Industr. Appl. Mothem* **1955**.
24. Press, W. H.; Teukolsky, S. A.; Vetterling, W. T.; Flannery, B. P. J. T. a. o. s. c., Numerical recipes in Fortran 77. *Cambridge University Press* **1992,** *1*.
25. Goldenberg, H.; Tranter, C., Heat flow in an infinite medium heated by a sphere. *British journal of applied physics* **1952,** *3* (9), 296-298.
26. Govorov, A. O.; Zhang, W.; Skeini, T.; Richardson, H.; Lee, J.; Kotov, N. A., Gold nanoparticle ensembles as heaters and actuators: melting and collective plasmon resonances. *Nanoscale Research Letters* **2006,** *1* (1), 84.
27. Baffou, G.; Berto, P.; Bermúdez Ureña, E.; Quidant, R.; Monneret, S.; Polleux, J.; Rigneault, H., Photoinduced heating of nanoparticle arrays. *ACS Nano* **2013,** *7* (8), 6478-6488.
28. Behrens, S. H.; Grier, D. G., The charge of glass and silica surfaces. *The Journal of Chemical Physics* **2001,** *115* (14), 6716-6721.
